# Src family kinases Fyn and Lyn are constitutively activated and mediate plasmacytoid dendritic cell responses

**DOI:** 10.1038/ncomms14830

**Published:** 2017-04-03

**Authors:** S. Dallari, M. Macal, M. E. Loureiro, Y. Jo, L. Swanson, C. Hesser, P. Ghosh, E. I. Zuniga

**Affiliations:** 1 Molecular Biology Section, Division of Biological Sciences, University of California, 9500 Gilman Dr La Jolla, San Diego, California 92093, USA; 2 Departments of Medicine and Cellular and Molecular Medicine, University of California, 9500 Gilman Dr La Jolla, San Diego, California 92093, USA

## Abstract

Plasmacytoid dendritic cells (pDC) are type I interferon-producing cells with critical functions in a number of human illnesses; however, their molecular regulation is incompletely understood. Here we show the role of Src family kinases (SFK) in mouse and human pDCs. pDCs express Fyn and Lyn and their activating residues are phosphorylated both before and after Toll-like receptor (TLR) stimulation. Fyn or Lyn genetic ablation as well as treatment with SFK inhibitors ablate pDC (but not conventional DC) responses both *in vitro* and *in vivo*. Inhibition of SFK activity not only alters TLR-ligand localization and inhibits downstream signalling events, but, independent of *ex-vivo* TLR stimulation, also affects constitutive phosphorylation of BCAP, an adaptor protein bridging PI3K and TLR pathways. Our data identify Fyn and Lyn as important factors that promote pDC responses, describe the mechanisms involved and highlight a tonic SFK-mediated signalling that precedes pathogen encounter, raising the possibility that small molecules targeting SFKs could modulate pDC responses in human diseases.

## Abstract (web summary)

Type I interferon responses are a core immunoregulatory function of plasmacytoid dendritic cells (pDCs). Here the authors show that SFK family members, including Lyn and Fyn, control type I interferon production in human and mouse pDCs.

Type I interferons (type I IFNs) are a family of innate mediators primarily involved in host defence against invading pathogens, which are sensed through pattern recognition receptors^1^,^2^,^3^. This cytokine family is composed of 13 partially homologous IFN-α subtypes, a single IFN-β subtype and several other poorly defined single gene products (IFN-ɛ, IFN-δ, IFN-ζ, IFN-κ and IFN-τ)^3^. Type I IFN production is initiated hours after infection and drives hundreds of interferon-stimulated genes that induce an antimicrobial state in infected and bystander cells, therefore limiting the spread of infectious agents in a cell-intrinsic manner^4^. In addition, type I IFNs orchestrate innate and adaptive immune cells, by promoting natural killer cell functions, antigen presentation and T-cell proliferation^3^. That mice lacking type I IFN receptor (IFNAR) are more susceptible to a wide range of infections and tumours are therefore not surprising^3^,^5^. By contrast, excessive production of type I IFNs is associated with several autoimmune diseases in mice and humans, including psoriasis and systemic lupus erythematosus^6^,^7^. Finally, persistently low production of type I IFNs can have immunosuppressive effects during chronic infections, highlighting the double-edged nature of these innate mediators^8^,^9^,^10^,^11^.

Although almost all cell types can produce type I IFNs, plasmacytoid dendritic cells (pDCs) secrete several hundred times more interferon subtypes than any other blood cell type upon Toll-like receptor (TLR) stimulation^12^,^13^,^14^. pDCs sense self or microbial single-stranded RNA or unmethylated CpG DNA via endosomal TLR7 and TLR9, respectively, triggering a signalling cascade that involves IKKα/β phosphorylation and induces type I IFNs (via interferon regulatory factor (IRF)-7) as well as pro-inflammatory cytokines and co-stimulatory molecules (via NF-κB)^2^. The unique type I IFN production capacity of pDCs is associated with constitutively high expression of IRF-7 as well as prolonged retention of TLR ligands in IRF-7-associated endosomal vesicles, which is critical for IRF-7 activation and type I IFN production^15^,^16^,^17^. Several surface receptors have been reported to fine-tune type I IFNs and pro-inflammatory cytokine production in pDCs. These receptors or their adaptors generally display an immunoreceptor tyrosine-based inhibition motif (ITIM) or an immunoreceptor tyrosine-based activation motif (ITAM), which function as docking sites for downstream signalling molecules^18^. ITAMs and ITIMs usually undergo tyrosine phosphorylation by Src family kinases (SFKs), a family of non-receptor tyrosine kinases involved in a wide range of biological processes, including immune responses^19^. Indeed, selected SFK family members are expressed in immune cell populations and have important regulatory functions^19^,^20^.

Given that ITAM/ITIMs are present in pDC regulatory receptors, which are often phosphorylated by SFKs, and that pan-SFKs inhibitors can both reduce pDC type I IFN production^21^,^22^ and reduce signal transduction from inhibitory receptors^23^,^24^, here we investigate the function that members of the SFK family have in pDC responses. We show that pDCs express several SFK family members, with Fyn and Lyn expression in both mouse and human pDCs. These two SFKs members are phosphorylated in their activating residue before and after TLR stimulation. Importantly, genetic deletion of Lyn or Fyn as well as treatment with the pan-SFK inhibitor PP2 or the selective dual BCR-ABL/LYN inhibitor bafetinib reduced mouse and human pDC responses to TLR agonists, including the production of type I IFNs and pro-inflammatory cytokines. Furthermore, Lyn- and Fyn-deficient mice have reduced pDC responses after *in vivo* murine cytomegalovirus (MCMV) infection. We show that inhibition of SFK activity alters TLR-ligand localization and impairs several important signalling events triggered by TLR stimulation, as well as TLR-ligand independent events such as B-cell adaptor for phosphoinositide 3-kinase (also known as BCAP or PIK3AP1) phosphorylation (an adaptor protein that bridges PI3K and TLR pathways). These results demonstrate that selected SFKs, such as Fyn and Lyn, are critical for central constitutive and TLR-induced signalling events, optimal TLR-ligand trafficking and functional responses in mouse and human pDCs, uncovering a tonic pDC-signalling pathway that involves these kinase activities and is in place before pathogen encounter.

## Results

### SFK activating phosphorylation is TLR stimulation independent

**Figure 1:**
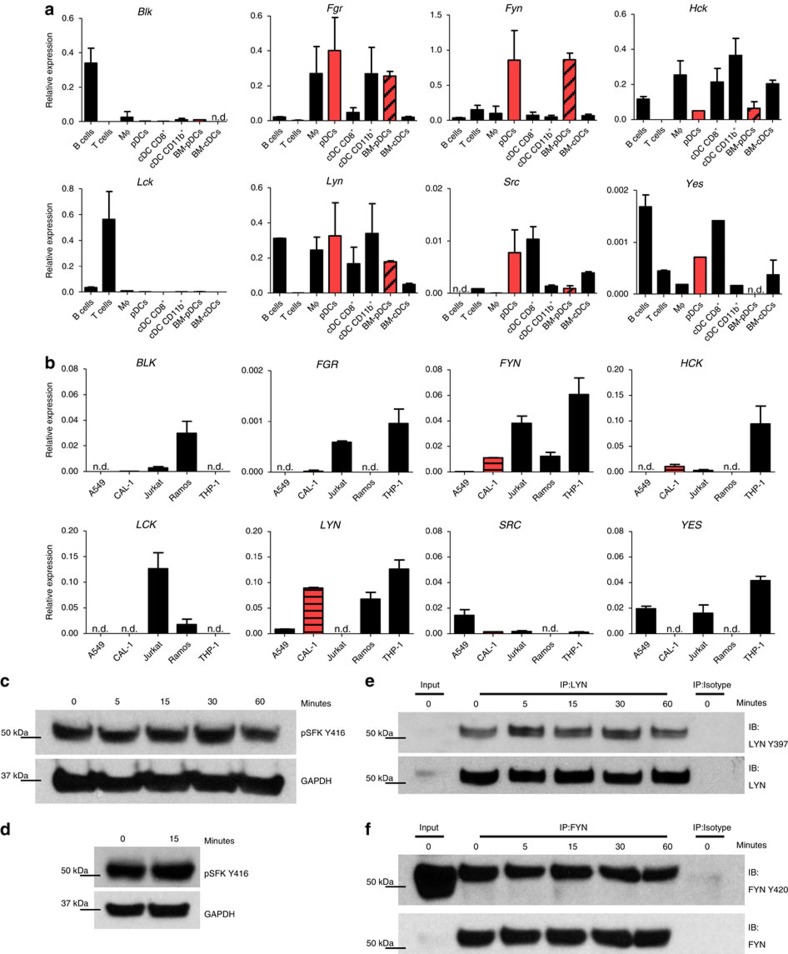
pDCs have constitutive LYN and FYN expression and activating phosphorylation. The expression of SFK mRNAs in mouse immune subsets (**a**) and human cell lines (**b**) were assessed by qPCR and normalized against the expression of GAPDH. In **a**, the immune subsets were FACS purified from freshly isolated spleen or from BM cells cultivated for 8 days in the presence of Flt3L. In **b**, cells from human cell lines were collected before reaching confluency and then total RNAs were extracted. Splenic pDCs are highlighted in solid red; BM-derived pDCs are highlighted in black-diagonal striped red; CAL-1 are highlighted in black-horizontal striped red. Graph bars represent mean±s.d. of two independent experiments. A549: lung epithelial cell line; CAL-1: human pDC cell line; Jurkat: T-cell line; Ramos: B-cell line; THP-1: monocyte cell line. (**c**,**d**) CAL-1 cells (**c**) or human primary pDCs (**d**) were stimulated with R848 for the indicated time periods (minutes) and SFK activating tyrosine phosphorylation (Y416) and GAPDH were assessed by immunoblot. (**e**,**f**) CAL-1 cells were stimulated with R848 for the indicated time periods (minutes) and protein immunoprecipitation was performed using anti-LYN (**e**) or anti-FYN (**f**) antibodies or isotype control antibody. Levels of LYN or FYN activating tyrosine phosphorylation and total LYN or FYN were assessed by immunoblot. Results are representative of two independent experiments (**c**–**e**,**f**) or three individual donors (**d**).

Every immune cell type expresses a selected set of SFKs, which contributes to their cell type-specific responses^19^. Up to now, SFK expression in pDCs has been assessed only for a few members, showing high *LYN* expression accompanied by low *FYN* levels in human pDCs^23^,^25^. To have an overall picture of all SFK members and their conservation between mouse and human pDCs, we first evaluated the transcript levels of SFK in mouse primary pDCs, FACS purified from spleen or BM-Flt3L cultures, and compared them with other immune cells. We observed that *Fgr*, *Fyn* and *Lyn* were expressed at relatively high levels in both splenic and bone marrow (BM)-derived mouse pDCs, with *Fyn* expression being several folds higher in pDCs than any other cell type (Fig. 1a). In contrast, *Blk*, *Hck*, *Lck*, *Src* and *Yes* were expressed at low levels. We next assessed SFKs in a human pDC cell line, CAL-1, which recapitulates many features of primary human pDCs^26^ (Fig. 1b). Similar to mouse pDCs and consistent with previous reports in human primary pDCs^23^,^25^, CAL-1 cells showed significant expression of *LYN*. However, unlike mouse pDCs, CAL-1 exhibited relatively low (although detectable) *FYN* expression and almost undetectable levels of *FGR*. *BLK*, *HCK*, *SRC*, *LCK* and *YES* also exhibited either low or undetectable expression.

We next analysed total SFK phosphorylation status before and after TLR stimulation. Unexpectedly, we observed that SFKs were active, as determined by autophosphorylated state of its catalytic domain that indicates full activation of the kinase^19^, even before TLR stimulation and that such activation was sustained for at least 60 min after incubation with the TLR7-ligand R848 (Fig. 1c). Remarkably, similar results were obtained with primary pDCs from human peripheral blood (Fig. 1d). Further analysis of specific LYN and FYN activating phosphorylation in CAL-1 cells revealed that both SFK members were phosphorylated before and after TLR stimulation (Fig. 1e,f). Together, these data indicated that both mouse and human pDCs express Lyn and Fyn kinases (albeit at different relative levels) and that these kinases were constitutively active irrespective of TLR activation.

### Fyn and Lyn mediate *in vitro* response to TLR stimulation

**Figure 2:**
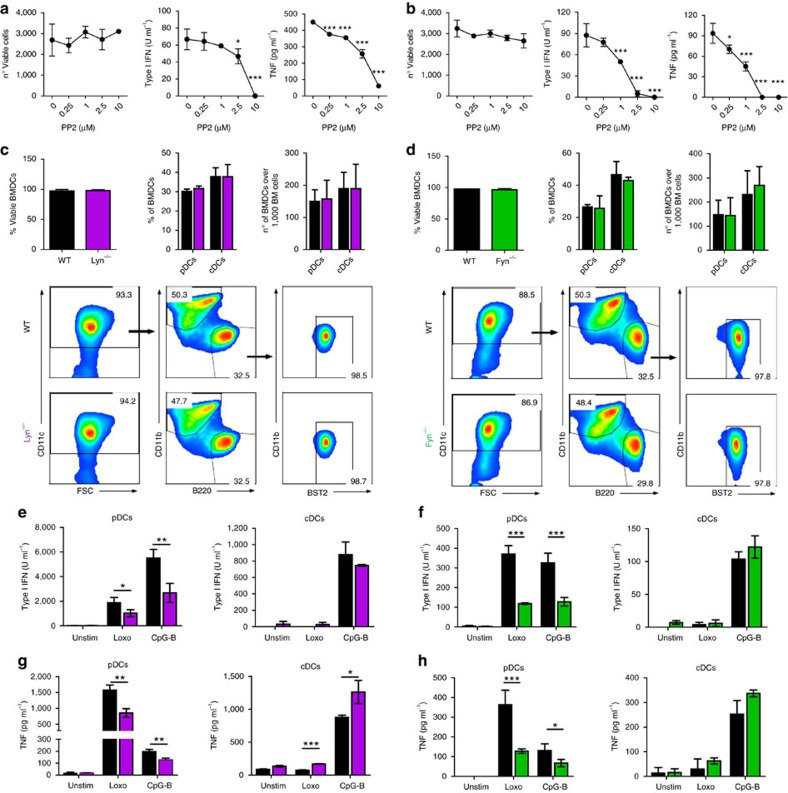
SFK members Lyn and Fyn are necessary for TLR-induced responses in mouse pDCs. (**a**,**b**) FACS-purified BM-derived pDCs were pre-treated with PP2 or DMSO control for 1 h and then stimulated with CpG-B (**a**) or loxoribine (**b**) for 15 h. Cell viability was assessed by flow cytometry, while type I IFN and TNF protein levels in the supernatant were assessed by bioassay and ELISA, respectively. (**c**–**h**) BM-Flt3L cultures derived from WT (black), Lyn^−/−^ (violet, **c**,**e**,**g**) or Fyn^−/−^ (green, **d**,**f**,**h**) were collected at day 8 post-culture. DC viability and development were assessed by flow cytometry (**c**,**d**). pDCs and cDCs were FACS purified and stimulated with CpG-B or loxoribine (loxo) for 15 h. Type I IFN activity (**e**,**f**) and TNF protein level (**g**,**h**) in the supernatant were assessed by bioassay and ELISA, respectively. Data are representative of two (**a**,**b**), three (**d**,**f**,**h**) or four out of five (**c**,**e**,**g**) independent experiments with *n*=2 pooled mice per group (**c**–**h**). Graphs depict mean±s.d. of replicates within one representative experiment. Dunnett's multiple comparisons test (**a**,**b**) and two-way Student's *t*-test (**c**–**h**) were used for statistical analyses. *P<0.05, **P<0.01, ***P<0.001.

Having confirmed a significant expression and activation of selected SFK members in pDCs, we assessed the role of SFKs in mouse pDC function, comparing it also side-by-side with cDCs. For this and to overcome redundant activity among different SFKs^20^, we treated FACS-purified BM-derived pDCs with the pan-SFK inhibitor PP2. While PP2 treatment did not significantly affect pDC viability, it dramatically decreased, in a dose-dependent manner, the production of both type I IFNs and tumour necrosis factor (TNF) by BM-derived pDCs and BMDCs stimulated with the TLR9-ligand CpG-A or -B (Fig. 2a and Supplementary Fig. 1a). Furthermore, upregulation of maturation molecules CD86 (and to lower extent MHC-II) in response to TLR9 stimulation was also compromised by PP2 treatment (Supplementary Fig. 1b). Similar results were obtained when TLR7-ligand loxoribine was used to stimulate pDCs in the presence or absence of PP2 (Fig. 2b and Supplementary Fig. 1c). In contrast, no significant decrease in cytokine production or CD86 was observed in PP2-treated cDCs (Supplementary Fig. 1d,e).

Given that mouse pDCs shared Lyn and Fyn expression with human pDCs (Fig. 1a,b), we next took advantage of Lyn^−/−^ and Fyn^−/−^ mice to evaluate the specific roles of these two SFKs in pDCs versus cDCs. To avoid indirect effects due to the altered environment in Lyn- or Fyn-deficient mice^27^, we first used pDCs generated after 7–8 days in BM cultures with Flt3L. As shown in Fig. 2c,d, neither cell viability nor the percentages or numbers of DC generated were altered in BM cultures from Lyn^−/−^ or Fyn^−/−^ mice compared to WT littermate controls. FACS-purified Lyn^−/−^ and Fyn^−/−^ pDCs, however, produced significantly lower levels of type I IFNs and TNF than WT pDCs after either CpG or loxoribine stimulations (Fig. 2e–h and Supplementary Fig. 1f), whereas no substantial differences in *Tlr7* and *Tlr9* mRNA expression were observed (Supplementary Fig. 1g). Instead, cDCs purified from Lyn^−/−^ or Fyn^−/−^ BM cultures did not show a decreased cytokine production, and instead exhibited an increase in TNF production by Lyn^−/−^ cDCs, which is consistent with previous findings (Fig. 2g and ref. 28). In contrast to overall SFK inhibition by PP2 treatment, no or minimal changes in TLR-induced upregulation of CD86 or MHC-II expression were observed in Lyn^−/−^ and Fyn^−/−^ pDCs and similar results were obtained with cDCs (Supplementary Fig. 1h–k). Taken together, our data showed that SFK Lyn and Fyn promoted TLR responses in mouse pDCs but not cDCs. Furthermore, the more profound effect of pan-SFK inhibition versus individual deletion of Lyn or Fyn suggested partly redundant roles of SFK members in promoting pDC functions.

### Fyn and Lyn promote pDCs response to *in vivo* MCMV infection

**Figure 3:**
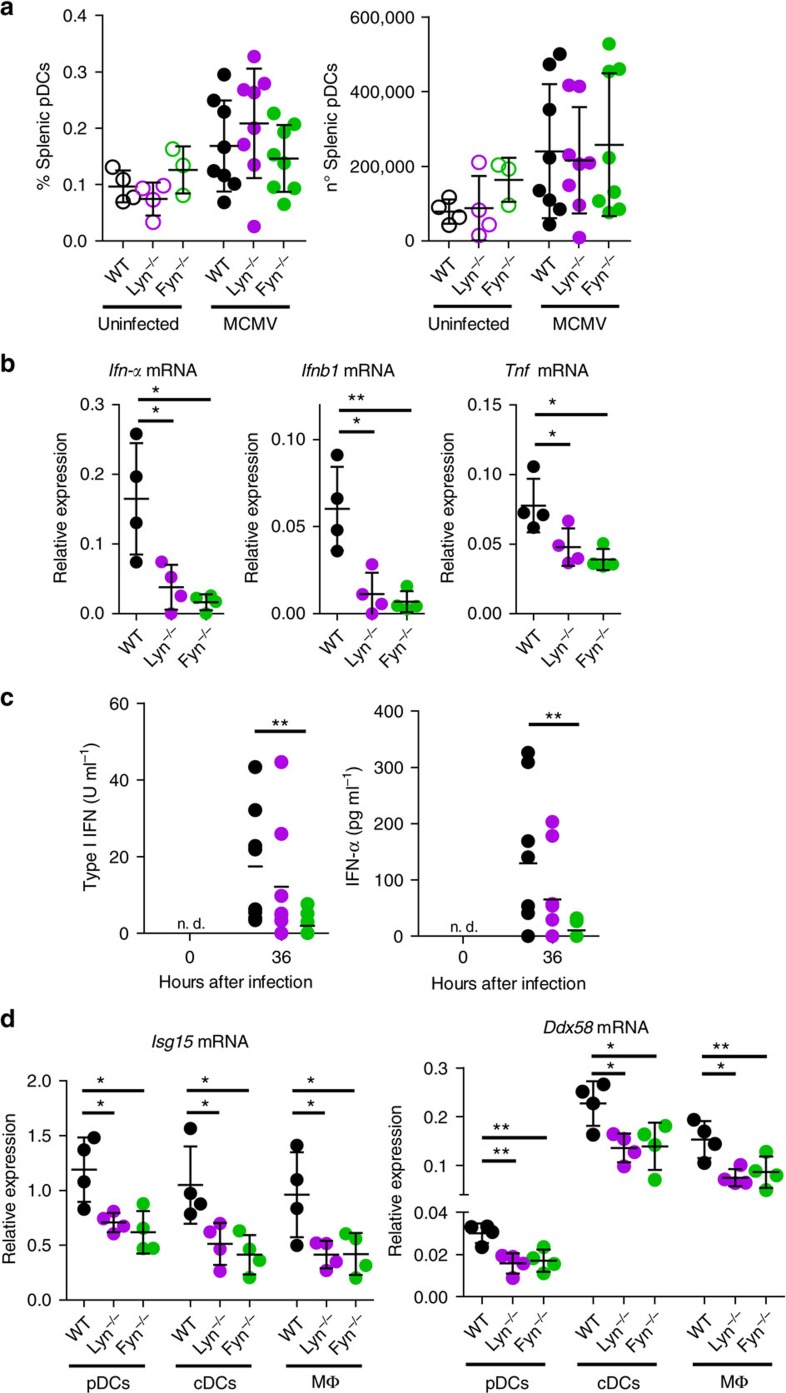
Fyn and Lyn promote pDC responses after *in vivo*viral infection. WT (black), Lyn^−/−^ (violet) or Fyn^−/−^ (green) mice were infected with MCMV for 36 h or left uninfected. (**a**) The proportion and number of pDCs were determined by FACS in spleen mononuclear cells. (**b**) The levels of *Tnf*, *Ifna* and *Ifnb1* were determined by q-PCR in FACS-purified splenic pDCs. (**c**) Serum IFN-α and type I IFNs were determined by ELISA and bioassay, respectively. (**d**) The levels of interferon-stimulated genes *Isg15* and *Ddx58* were determined by q-PCR in FACS-purified splenic pDCs, CD11b^+^ cDCs and macrophages. (**a**–**d**) Data were obtained in two independent experiments with four mice per group each. Mean value alone or mean value±s.d. are shown. Each symbol represents an individual mouse (**a**,**c**) or two pooled mice (**b**,**d**). Kruskal–Wallis test (**a**,**c**) and Dunnett's multiple comparisons test (**b**,**d**) were used for statistical analyses. *P<0.05, **P<0.01, ***P<0.001.

To rule out the possibility that the aforementioned SFK role in pDCs could result from a cell culture artifact or synthetic TLR agonists, we next examined the relevance of the SFK members Fyn and Lyn in pDC and type I IFN responses after an *in vivo* viral infection in its natural host. For that, we challenged WT, Lyn^−/−^ and Fyn^−/−^ mice with MCMV and we analysed pDCs 36 h post-infection, when they exhibit peak responses^29^,^30^. Consistently with the lack of SFK effects on pDC viability and development (Fig. 2c,d), Lyn^−/−^ and Fyn^−/−^ mice showed similar pDC proportions and numbers compared to WT mice both before and after MCMV infection (Fig. 3a and Supplementary Fig. 2a). In contrast, expression of *Tnf*, *Ifna* and *Ifnb1* were profoundly decreased in FACS-purified pDCs from Lyn^−/−^ and Fyn^−/−^ mice compared to WT controls after MCMV infection (Fig. 3b). As expected, *Ifna* and *Ifnb1* transcript levels were undetectable in pDCs from Fyn^−/−^, Lyn^−/−^ or WT uninfected mice (Supplementary Fig. 2b). Of note, *Ifna* and *Ifnb1* were undetectable or at background levels in both cDCs and macrophages from WT, Lyn^−/−^ and Fyn^−/−^ mice at 36 h post-MCMV infection (Supplementary Fig. 2c,d). Furthermore, in line with the results from BM cultures (Supplementary Fig. 1e–h), we observed similar expression of CD86 and MHC-II in pDCs from Lyn^−/−^, Fyn^−/−^ or WT mice before or after MCMV infection (Supplementary Fig. 2e).

Consistently with the decreased pDC type I IFN induction, systemic type I IFN bioactivity and IFN-α protein levels appeared reduced in Lyn^−/−^ and Fyn^−/−^ MCMV-infected mice versus their WT counterparts, although differences did not reach statistical significance for the Lyn^−/−^ group (Fig. 3c). Importantly, expression of *Isg15* and *Ddx58*, two type I IFN-stimulated genes, were significantly diminished in pDCs, cDCs and macrophages purified from either Lyn^−/−^ or Fyn^−/−^ mice versus WT controls after MCMV infection (Fig. 3d), suggesting that compromised pDC responses limited type I IFN availability for many cell types in the infectious environment. Taken together, our data indicated that SFK members Fyn and Lyn were critical for optimal pDC cytokine production and type I IFN responses after an *in vivo* viral infection in its natural host.

### SFKs mediate human pDC response to TLR ligand

**Figure 4:**
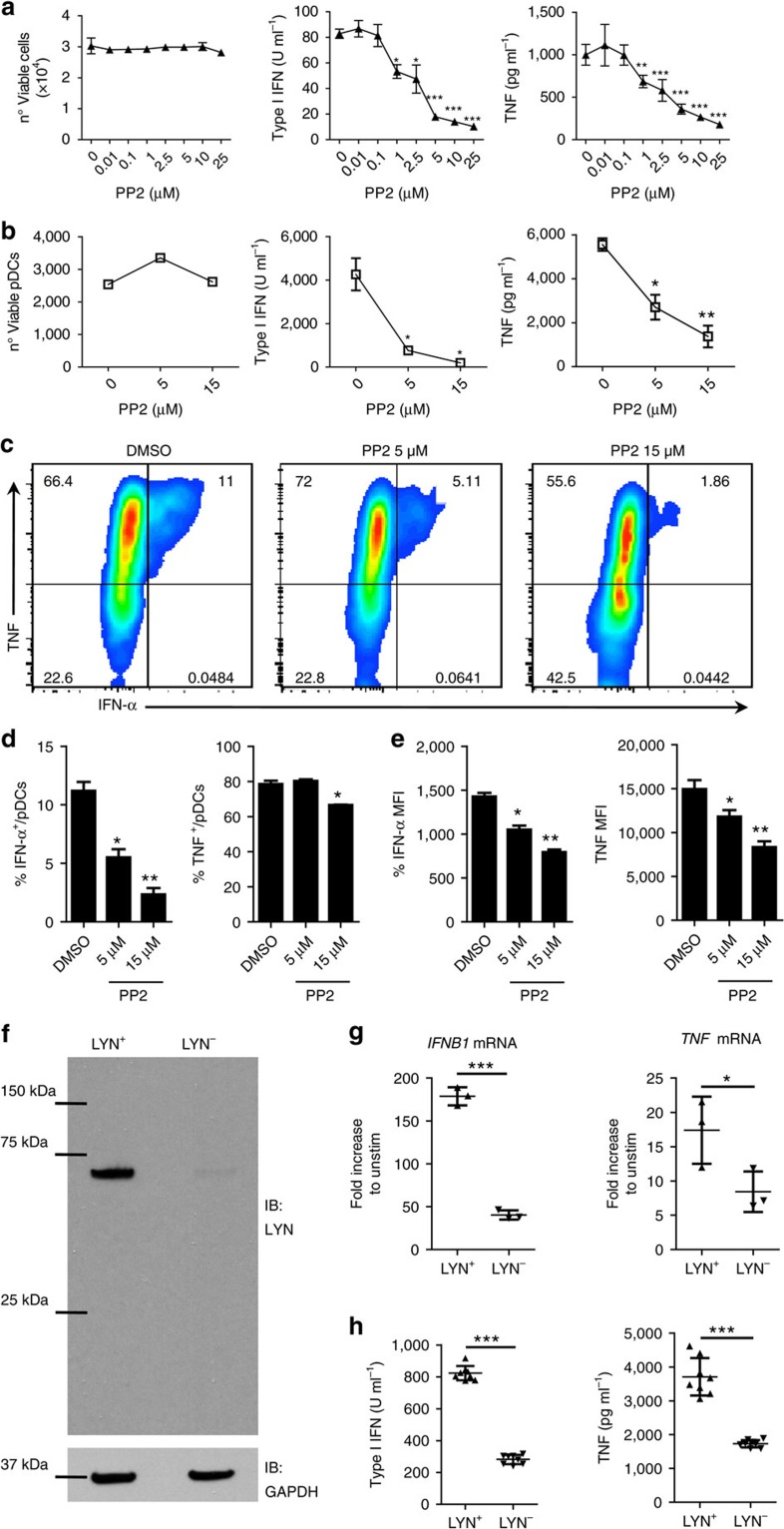
SFKs are critical for human pDC cytokine production. (**a**,**b**) CAL-1 cells (**a**) or human PBMCs (**b**) were pre-treated for 1 h with PP2 or DMSO control and then stimulated with R848 for 15 h. Viability was assessed by flow cytometry, while type I IFN and TNF protein levels in the supernatant were assessed by bioassay and ELISA, respectively. (**c**–**e**) Human PBMCs were pre-treated for 1 h with PP2 (5 and 15 μM) or DMSO control and then stimulated with R848 for 6 h and subsequently intracellular stained for TNF and IFN-α. FACS plots show the co-expression of TNF and IFN-α in gated pDCs (**c**). The percentage of pDCs single positive to IFN-α and TNF staining (**d**). MFI of IFN-α and TNF in gated pDCs (**e**). (**f**) LYN and GAPDH protein expression in unmodified CAL-1 cells (LYN^+^) and CAL-1 cells ablated for LYN by CRISPR/Cas9 (LYN^−^) was determined by immunoblot. (**g**,**h**) The expression of IFN-β and TNF mRNAs was determined by qPCR (**g**) and the level of protein in the supernatant was quantified by bioassay or ELISA (**h**) in LYN^+^ and LYN^−^ CAL-1 cells after 1 or 15 h stimulation with R848, respectively. In **g**, the mRNA expression were normalized against GAPDH expression and plotted as mRNA fold increase compared to unstimulated cells. Data are representative of two (**g**) or three (**a**,**f**,**h**) independent experiments or four donors processed separately (**b**–**e**). Graphs depict mean±s.d. of replicates within one representative experiment/donor. Dunnett's multiple comparisons test (**a**,**b**,**d**,**e**) and two-ways Student's *t*-test (**f**,**g**) were used for statistical analyses. *P<0.05, **P<0.01, ***P<0.001.

While mouse pDCs recapitulate most functional and molecular features of human pDCs, several differences in regulatory receptors have been previously identified^18^. To validate and expand previous studies^21^,^22^ on the overall SFK role in human pDCs, we first determined type I IFNs and TNF levels after stimulating CAL-1 cells with the TLR7/8 agonist R848 in the presence of the pan-SFK inhibitor PP2. Similarly to mouse pDCs, CAL-1 cells showed comparable viability but a significant reduction in both type I IFN and TNF production when treated with increasing doses of PP2 (Fig. 4a). Importantly, the profound PP2-induced cytokine suppression in CAL-1 cells was recapitulated with pDCs from human peripheral blood obtained from healthy donors (Fig. 4b). Furthermore, PP2 treatment caused a decrease in the percentage of positive pDCs producing type I IFNs and/or TNF at the single cell level (Fig. 4c,d and Supplementary Fig. 3a). While type I IFN production appeared more sensitive to PP2 inhibitory effect than TNF production, which only showed reduced proportion of cytokine producing pDCs at the highest dose tested, TNF median fluorescence intensity (MFI) was significantly affected at both 5 and 15 μM PP2 concentrations (Fig. 4d,e).

Given that SFK transcripts indicated that Lyn is the most abundant SFKs in the human pDC cell line (Fig. 1b), we next used CRISPR/Cas9 technology to genetically ablate *LYN* exon 4 in CAL-1 cells (Supplementary Fig. 4). This approach resulted in almost complete loss of the LYN protein expression, with no evidence of truncated LYN variants (Fig. 4f). Importantly, the absence of LYN in CAL-1 cells profoundly compromised *IFNB1* and *TNF* mRNA induction as well as type I IFN activity and TNF protein levels after TLR7/8 stimulation (Fig. 4g,h). Overall, these data support the critical role of SFKs in mediating human pDC cytokine production upon TLR stimulation and identified LYN as one SFK member partly responsible for such effect.

### Central TLR signalling event activation requires SFK activity

**Figure 5:**
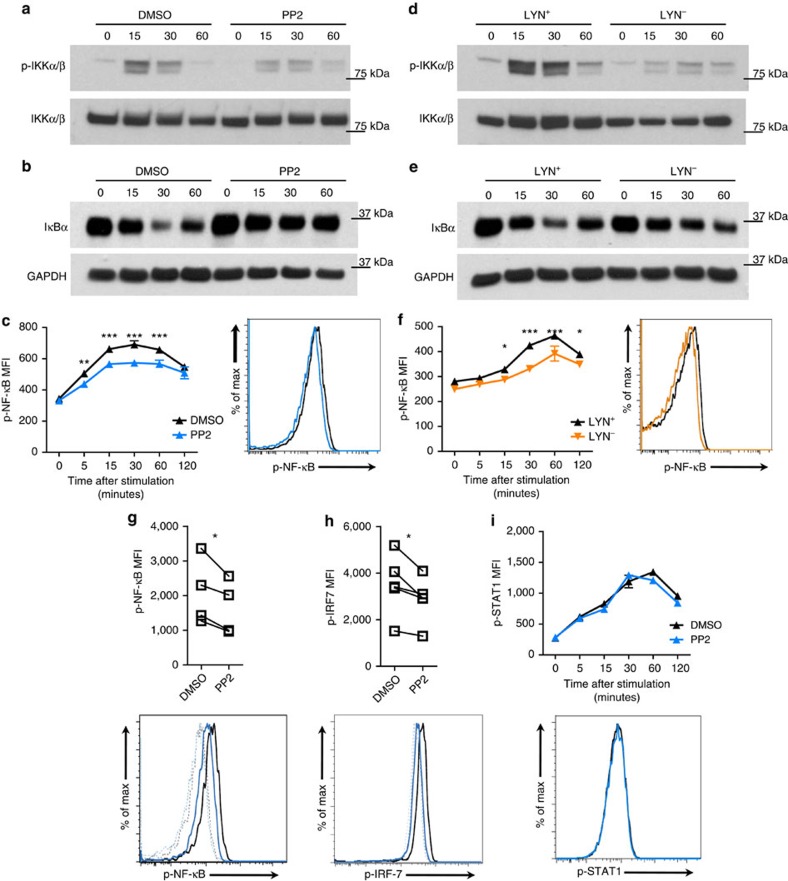
SFKs are necessary for TLR signalling events in human pDCs. (**a**–**f**) WT or LYN^−^ (**d**–**f**) CAL-1 cells were pre-treated for 1 h with PP2 (10 μM; **a**–**c**; blue) or DMSO control (**a**–**c**; black) or left untreated (**d**–**f**; black: LYN^+^, orange: LYN^−^), before stimulation with R848 for the indicated time periods (minutes). Level of p-IKKα/β and total IKKα/β (**a**,**d**) as well as IκBα (**b**,**e**) and GAPDH were assessed by immunoblot. p-NF-κB was determined by flow cytometry. The histogram depicts the 15 min time point (**c**,**f**). (**g**,**h**) Human PBMCs were pre-treated as in **c** and stimulated for 15 min with R848. p-NF-κB (**g**) and p-IRF-7 (**h**) were determined by flow cytometry in gated pDCs. Lines in graphs connect the same donor and histograms depict one representative donor; dotted lines, unstimulated; solid lines, R848 stimulation. (**i**) CAL-1 cells were pre-treated as in **c** before stimulation with recombinant IFN-β (1,000 U ml^−1^) for the indicated time periods (minutes). p-STAT1 were determined by flow cytometry. Histogram depicts the 30 min time point. Data are representative of two (**b**,**e**,**f**,**i**) or three (**a**,**c**,**d**) independent experiments and four (**g**) or five (**h**) donors processed separately. Graphs depict mean±s.d. of replicates within one representative experiment (**a**–**f**,**i**) or individual donors (**g**,**h**). Two-way ANOVA (**c**,**f**,**i**) and two-ways Student's *t*-test (**g**,**h**) were used for statistical analyses. *P<0.05, **P<0.01, ***P<0.001.

TLR7 and TLR9 signal through similar pathways, which involve the phosphorylation and ubiquitination of kinases and adaptor proteins, leading to the activation of several key transcription factors, such as NF-κB and IRF-7, that induce cytokine gene transcription^1^,^2^. To understand the aforementioned SFK role in pDC cytokine production, we next investigated SFK regulation of pDCs at the molecular level. We first evaluated phosphorylation of IKKα/β, the kinase complex involved in both NF-κB and IRF-7 activation^2^. As expected, CAL-1 cells treated with DMSO rapidly increased IKKα/β phosphorylation 15 min after R848 stimulation, returning to basal levels by 60 min after stimulation (Fig. 5a). Notably, the phosphorylation of IKKα/β was significantly reduced in PP2-treated CAL-1 cells at both 15 and 30 min after TLR stimulation while the levels of total IKKα/β remained unchanged (Fig. 5a). Consistently, R848-mediated IκBα degradation, NF-κB phosphorylation induction and nuclear translocation were decreased in PP2-treated versus DMSO-treated CAL-1 cells (Fig. 5b,c and Supplementary Fig. 5a). Furthermore, both IKKα/β and NF-κB activation and IκBα degradation were also reduced in Lyn-deficient CAL-1 cells compared to WT controls (Fig. 5d–f and Supplementary Fig. 5b), indicating a specific role for LYN early after TLR stimulation. Importantly, we could also detect a significant reduction in the phosphorylation of NF-κB and IRF-7 15 min after TLR stimulation of human primary pDCs pre-incubated with PP2 versus DMSO (Fig. 5g,h and Supplementary Fig. 3b). TLR7/9 activate also the mitogen-activated protein kinase p38 pathway, leading to phosphorylation and activation of members of the AP-1 family of transcription factors^1^,^2^. Assessment of p38 phosphorylation in DMSO-treated CAL-1 cells showed an overall threefold upregulation, reaching maximal levels at 30 min, after TLR stimulation. In comparison, the phosphorylation levels of p38 were significantly reduced in both PP2-treated and Lyn-deficient CAL-1 cells (Supplementary Fig. 5c,d) but this effect was not recapitulated with PP2 treatment in human primary pDCs analysed 30 min after TLR stimulation (Supplementary Fig. 5e). Type I IFN production by pDCs is strongly driven by type I IFN-positive feedback loop^31^,^32^. To assess the role of SFKs in the cytokine production driven by the type I IFN-positive feedback, we analysed STAT1 phosphorylation in CAL-1 cells, which is induced downstream IFNAR, following treatment with recombinant IFN-β. We observed comparable increase in STAT1 phosphorylation between DMSO-treated and PP2-treated CAL-1 cells (Fig. 5i), suggesting that SFKs are not required for IFNAR signalling or type I IFN-positive feedback loop. Taken together, these data indicated that SFKs were required for optimal TLR signalling in pDCs, impacting key molecular events such as IKKα/β, NF-κB and possibly IRF-7 phosphorylation minutes after TLR engagement.

### SFKs mediate MTOR activation and BCAP basal phosphorylation

**Figure 6:**
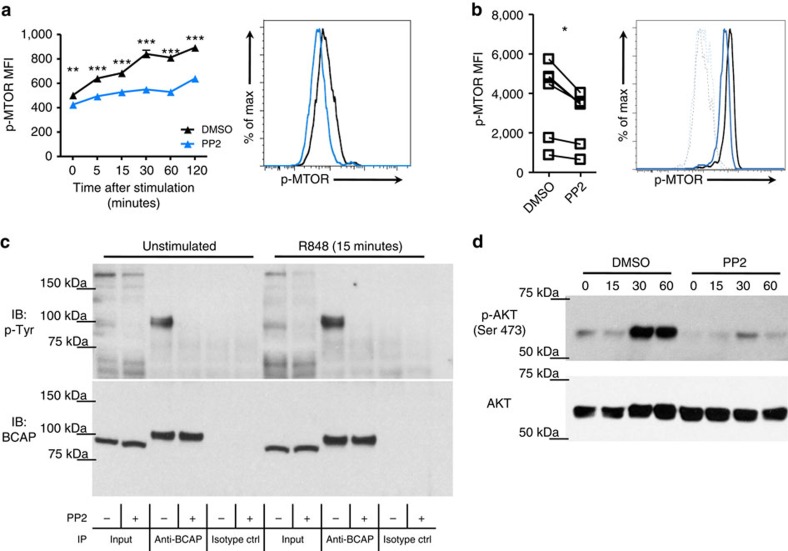
SFKs are necessary for mTOR and BCAP phosphorylation in human pDCs. (**a**) CAL-1 cells were pre-treated for 1 h with PP2 (10 μM; blue) or DMSO control (black) before stimulation with R848 for the indicated time periods (minutes). p-MTOR was determined by flow cytometry. Histograms depict the 15 min time point. (**b**) Human PBMCs were pre-treated as in **a** and stimulated for 15 min with R848. p-MTOR was determined by flow cytometry in gated pDCs. Lines in graph connect the same donor and the histogram depicts a representative donor; dotted lines, unstimulated; solid lines, R848 stimulation. (**c**) CAL-1 cells were pre-treated as in **a** and then stimulated with R848 for 15 min. Protein immunoprecipitation was performed using anti-BCAP antibody or isotype control antibody and levels of tyrosine phosphorylation (p-Tyr) and BCAP were assessed by immunoblot. (**d**) CAL-1 cells were pre-treated as in **a** and then stimulated with R848 for the indicated time periods (minutes). p-AKT Ser473 and AKT levels were determined by immunoblot. Data are representative of two (**d**) or three (**a**,**c**) independent experiments or five donors processed separately (**b**). Graphs depict mean±s.d. of replicates within one representative experiment (**a**,**c**,**d**) or individual donors (**b**). Two-way ANOVA (**a**) and two-ways Student's *t*-test (**b**) were used for statistical analyses. *P<0.05, **P<0.01, ***P<0.001.

Recent studies have revealed a critical role for PI3K^33^ and mechanistic target of rapamycin (MTOR)^34^ to enable pDC type I IFN production. To investigate if SFK activity could impact MTOR activation, we analysed MTOR phosphorylation level at Ser-2448 residue, which is critical for this kinase activation. As expected, CAL-1 cells upregulated MTOR phosphorylation within 5 min after TLR stimulation and for at least 120 min (Fig. 6a and Supplementary Fig. 6a). Remarkably, SFK inhibition induced by PP2 treatment resulted in a dramatic reduction in MTOR Ser-2448 phosphorylation at all time points studied after R848 stimulation (Fig. 6a). Importantly, we could also detect reduced MTOR activation 15 min after R848 stimulation in human blood pDCs that have been pre-treated with PP2 versus DMSO (Fig. 6b). These data indicated that SFK activity was necessary for TLR-mediated MTOR activation.

Recently, two studies have identified BCAP as the protein mediating the cross-talk between the PI3K-AKT-MTOR pathway and the TLR pathway^35^,^36^. This adaptor protein interacts with MYD88, through a Toll-IL-1 receptor domain, and with the regulatory subunit of PI3K, through a YxxM motif when the tyrosine in it is phosphorylated. It is still unclear which kinase phosphorylates BCAP, but different studies have proposed a role for Lyn, Syk and Btk as potential candidates^37^,^38^. Furthermore, *BCAP* transcript has been shown to be highly expressed by human pDCs^23^. To assess whether SFKs are involved in BCAP phosphorylation in pDCs, cell lysates from PP2- and DMSO-treated CAL-1 were subjected to immunoprecipitation with anti-BCAP antibody and analysed by immunoblot to detect tyrosine phosphorylation. Strikingly, we observed constitutive tyrosine phosphorylation signal at 90 kDa (BCAP molecular weight) that was significantly increased by BCAP immunoprecipitation in DMSO-treated CAL-1 cells under unstimulated conditions and showed no apparent changes after 15 min incubation with R848 stimulation (Fig. 6c, DMSO lanes). Remarkably, while there was no overall decrease in tyrosine phosphorylation when CAL-1 cells were pre-treated with PP2 (Supplementary Fig. 6b), BCAP tyrosine phosphorylation was completely ablated both before and after TLR stimulation (Fig. 6d, PP2 lanes). Consistently, AKT phosphorylation in Ser473, which is TLR- and PI3K-dependent in pDCs^33^ and secondary to BCAP engagement in macrophages^35^,^36^, was strongly suppressed when SFK activity was inhibited by PP2 (Fig. 6c). Altogether, these data indicated that SFK activity was essential for basal BCAP phosphorylation in pDCs, which would allow docking of PI3K to MYD88 (ref. 36), and optimal activation of AKT-MTOR axis after TLR stimulation.

### TLR-ligand intracellular localization requires SFKs in pDCs

**Figure 7:**
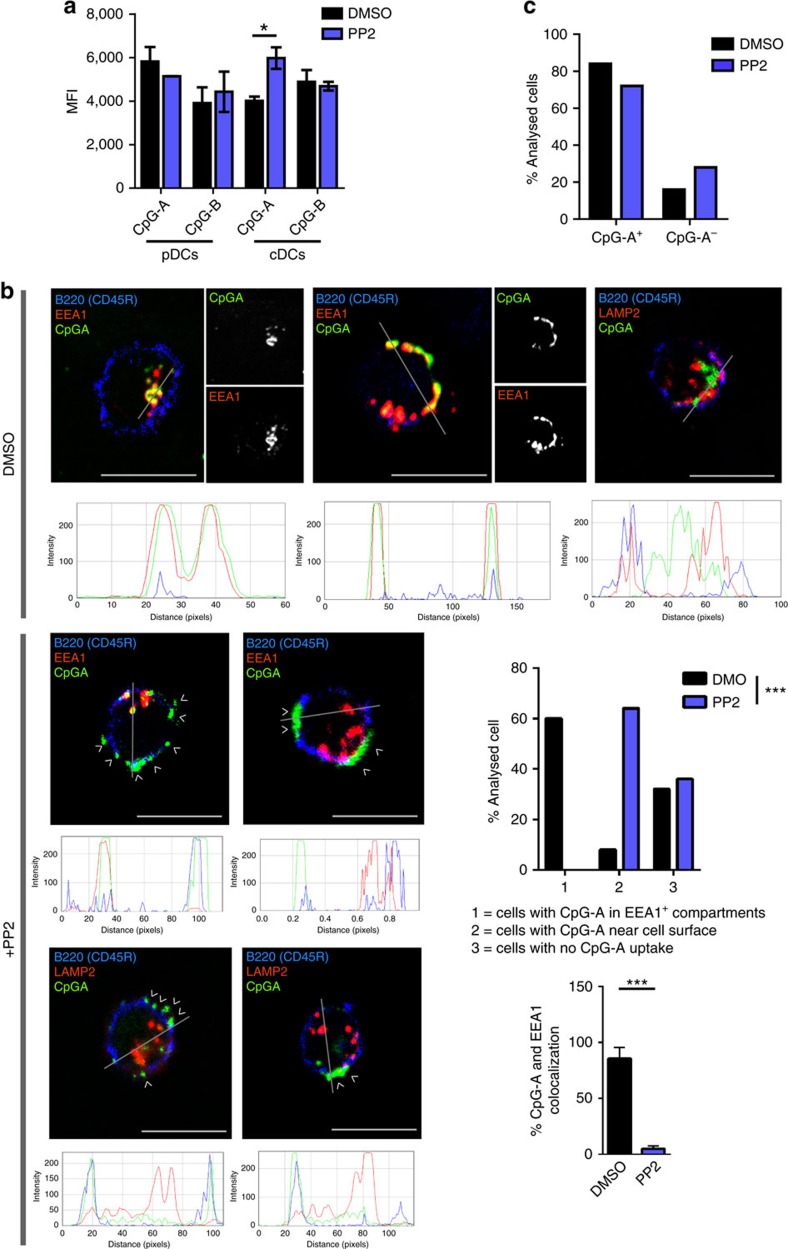
SFK activity is necessary for TLR-ligand localization in early endosomes of pDCs. (**a**) BM-derived DCs cells were pre-treated for 1 h with PP2 (10 μM; blue) or DMSO control (black) before stimulation with either CpG-A-FITC or CpG-B-FITC for 1 h. CpG uptake was determined by flow cytometry in gated cDCs and pDCs. pDCs were gated as CD11c^+^/CD11b^−^/BST2^+^. Data are representative of three independent experiments. Graphs depict mean±s.d. of replicates within one representative experiment. (**b**,**c**) BM-derived DCs were pre-treated for 1 h with PP2 (10 μM; blue) or DMSO control (black), before stimulation with CpG-A-FITC for 90 min. CpG-A-FITC internalization and colocalization with EEA1 or LAMP2 was evaluated by confocal microscopy in B220^+^ (**b**, pDCs) and B220^−^ (**c**, cDCs) cells. Examples of representative images are shown, with RGB plots underneath each panel. These plots quantify the degree of colocalization of CpG-A-FITC (green) with EEA1/LAMP2 (red) along the arbitrary line on each corresponding image, as measured using ImageJ. Bar graphs display the patterns of CpG-A-FITC localization/uptake and % colocalization with EEA1 determined in 50 cells analysed in two independent experiments (**b**). CpG uptake in cDCs is graphed as percentage of positive cells within 50 cells analysed in two independent experiments (**c**). Two-ways Student's *t*-test (**a**,**b**) and χ^2^-test (**b**,**c**) were used for statistical analyses. Scale bars, 10 μm. *P<0.05, **P<0.01, ***P<0.001.

Previous studies have highlighted that TLR signalling and ligand trafficking are closely intertwined and that localization of ligand to the compartment where it encounters the TLR is a key determinant of downstream signalling^17^,^39^,^40^. We hypothesized that the broad defects in the TLR and AKT-MTOR signalling pathways we observed upon SFK inhibition could be due to impaired ligand uptake/internalization. To test this hypothesis, we administered fluorescent-conjugated CpG-A and CpG-B to BM-derived DCs in the presence or absence of PP2. As shown in Fig. 7a and Supplementary Fig. 7a, PP2 administration did not affect overall CpG-A or CpG-B uptake in neither pDCs nor cDCs. We next evaluated the localization of fluorescent-conjugated CpG in early endosomes or lysosomes, as determined by organelle markers EEA1 and LAMP2, respectively. As previously reported^17^, CpG-A localized to EEA1 organelles in pDCs versus LAMP2 organelles in cDCs 90 min after administration (Fig. 7b and Supplementary Fig. 7b; upper panels). Remarkably, we found that while inhibition of SFKs with PP2 did not alter CpG-A trafficking to the lysosomes in cDCs (Fig. 7c and Supplementary Fig. 7b), it did impair CpG-A trafficking to EEA1^+^-early endosomes in pDCs (Fig. 7b). It is noteworthy that CpG-A in PP2-treated pDCs neither localized to LAMP2^+^ lysosomes and instead appeared to show a peripheral accumulation (Fig. 7b).

Together, these data indicated that SFK activity was necessary for TLR-ligand localization to early endosomes in pDCs, but largely dispensable for its trafficking into lysosomes in cDCs, suggesting differential SFK requirement for optimal TLR-ligand localization in pDCs versus cDCs.

### Bafetinib strongly suppresses pDC cytokine production

**Figure 8:**
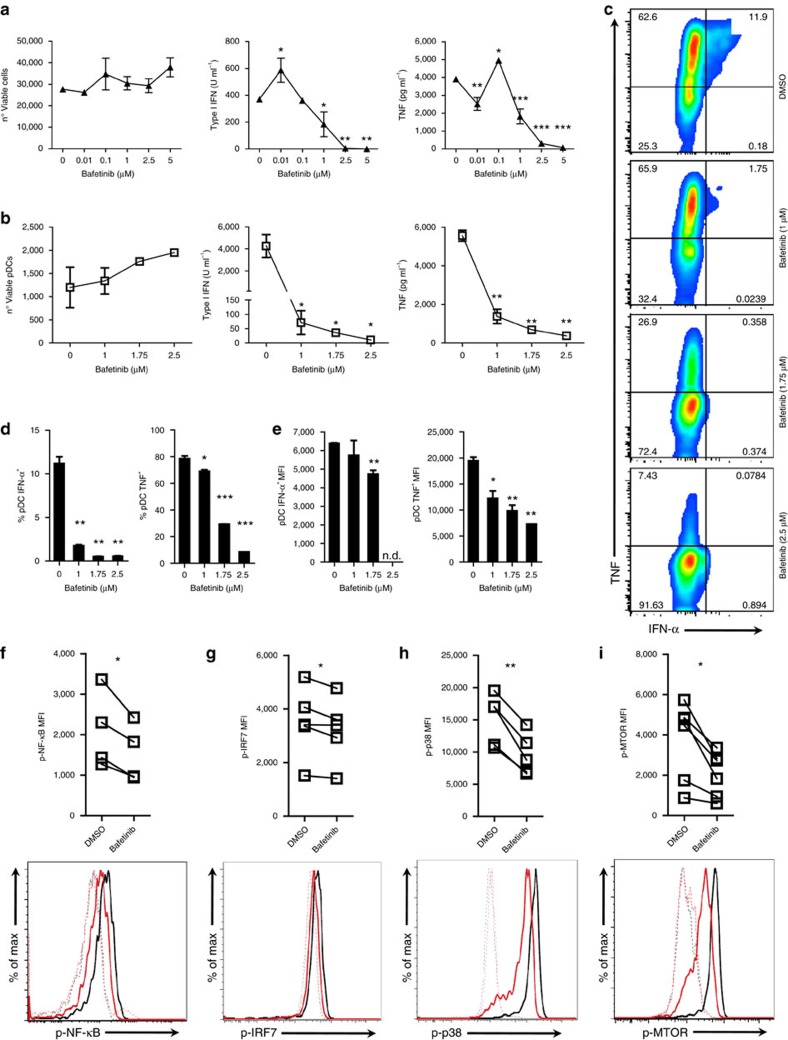
Bafetinib inhibits TLR-induced cytokine production and signalling in human pDCs. CAL-1 cells (**a**) or human PBMCs (**b**–**i**) were pre-treated for 1 h with bafetinib (5 μM) or DMSO control and then stimulated with R848. (**a**,**b**) Viability was assessed by flow cytometry, while type I IFN and TNF protein levels in the supernatant were assessed by bioassay and ELISA, respectively, at 15 h post-stimulation. (**c**,**d**) TNF and IFN-α were determined by flow cytometry at 6 h post-stimulation. Co-expression of TNF and IFN-α in gated pDCs (**c**). Percentage of pDCs single positive to IFN-α and TNF staining (**d**). MFI of IFN-α and TNF in gated IFN-α^+^ and TNF^+^ pDCs (**e**). (**f**–**i**) The level of p-NF-κB (**f**), p-IRF-7 (**g**), p-p38 (**h**) and p-MTOR (**i**) were assessed by flow cytometry in gated pDCs at 15 (**f**,**g**,**i**) or 30 (**h**) min after stimulation. Lines in graph connect the same donor and histograms depict a representative donor; dotted lines, unstimulated; solid lines, R848 stimulation; black line, DMSO; red line, bafetinib treated. Data are representative of five independent experiments (**a**) or four (**b**–**f**) and five (**g**–**i**) donors processed separately. Graphs depict mean±s.d. of replicates within one representative experiment (**a**–**e**) or individual donors (**f**–**i**). Dunnett's multiple comparisons test (**a**,**b**) and two-ways Student's *t*-test (**d**–**i**) were used for statistical analyses. *P<0.05, **P<0.01, ***P<0.001.

Bafetinib is a dual BCR-ABL/LYN inhibitor developed as a second-generation therapy for Philadelphia-positive leukaemia^41^ with phase I clinical trials demonstrating its safety for human administration^42^,^43^. This small molecule binds the amino acids of the target kinase ATP-binding site, stabilizing a non-ATP-binding configuration and preventing its activity^41^. Other than the chimeric protein BCR-ABL, typical of Philadelphia-positive leukaemia, bafetinib also inhibits a limited set of SFKs, including LYN and some FYN isoforms^41^. Based on these considerations, we next assessed the effect of bafetinib on CAL-1 and human blood pDC cytokine production after TLR stimulation. Interestingly, while bafetinib did not affect cell viability, it did cause a strong and dose-dependent inhibition of both type I IFN and TNF production by CAL-1 cells stimulated with R848, which became most apparent at bafetinib concentrations above 1 μM (Fig. 8a). Most remarkably, we observed that, in the absence of any effects on cell viability, bafetinib profoundly reduced the type I IFN and TNF production by human peripheral blood mononuclear cells (PBMCs) to almost undetectable levels (Fig. 8b). Furthermore, FACS analysis in human PBMCs revealed that bafetinib treatment caused a dose-dependent decrease in the percentage of type I IFN and TNF producing pDCs (Fig. 8c,d) as well as their corresponding MFI within cytokine producing cells (Fig. 8e). Finally, we found that bafetinib caused a profound reduction in NF-κB, IRF-7, p38 and MTOR phosphorylation in both CAL-1 cells and human blood pDCs upon TLR stimulation, with the degree of inhibition appearing even more profound than the aforementioned PP2 effects (Fig. 8f–i and Supplementary Fig. 8a–c). As with PP2, we could not detect any effect of bafetinib in STAT1 phosphorylation induced by recombinant IFN-β incubation (Supplementary Fig. 8d). These data demonstrated that bafetinib, a small molecule redeemed safe for human usage in a phase I clinical trial, profoundly suppressed early events in TLR signalling and the downstream cytokine production in human pDCs.

## Discussion

Type I IFN production by pDCs plays important roles in several disease contexts^3^,^6^,^11^,^14^,^44^,^45^. Therefore, pDC type I IFN production must be carefully regulated to ensure an effective immune response but at the same time avoid a breach in self-tolerance and immunopathology. In this study, we identified SFK members Fyn and Lyn as positive regulators of TLR signalling and functional responses in pDCs, the most powerful type I IFN producing cells.

By analysing expression of all SFK family members in pDCs versus other immune cell subsets, we demonstrated that *Fyn* expression in mouse pDCs was several folds higher than in other immune cells while *Lyn* was detected at significant levels in both mouse pDCs and the human pDC cell line CAL-1. These findings are consistent with previous studies showing that human pDCs express low FYN and high LYN levels^23^,^25^. Notably, activating phosphorylation of total SFKs, and specifically of FYN and LYN, was present in CAL-1 cells and human primary pDCs even before TLR stimulation. This observation is consistent with previous work in macrophages and CAL-1 cells showing that SFK activity is upstream of endosomal TLR^21^,^22^. These studies, however, detected SFK phosphorylation or activity after CpG stimulation. Thus, our data bring up a novel concept that tonic signals actively prime pDCs via SFK phosphorylation under steady-state conditions (before microbial encounter).

By using a combination of experimental approaches, including chemical inhibition and genetic ablation, we uncovered positive role for selected SFK members Fyn and Lyn in type I IFNs and pro-inflammatory cytokine production induced after *in vivo* viral infection or TLR stimulation in both mouse and human pDCs. Given that single Lyn or Fyn deficiency only partially reduced type I IFN and TNF production while the pan-SFK inhibitor PP2 almost completely ablated pDC responses, it is likely that several SFK members are partially redundant in pDCs. Our results are in line with previous reports showing reduced type I IFN levels when human pDCs were treated with SFK inhibitors^21^,^22^. Together with other two previous studies demonstrating that SFKs are necessary for calcium influx downstream BDCA-2, which negatively regulates TLR-induced type I IFN responses in pDCs^23^,^24^, these results indicate that SFKs play opposing roles in pDC cytokine regulation, being on one hand necessary for TLR-induced responses and on the other hand required for inhibitory signalling downstream BDCA-2. Such opposing roles by SFK have been previously described in multiple cell types, such as B cells, where SFKs activate ITAM downstream the BCR to promote their activation and ITIM downstream CD22 to inhibit their responses^46^,^47^. Similarly, Lyn can either boost or dampen the mast cell response to IgE according to the intensity of the stimuli^48^.

The positive Lyn role in pDC responses, that we observed both after TLR stimulation and *in vivo* natural MCMV infection, appears conflicting with the observations by Lamagna *et al*.^28^ showing a negative role for Lyn after TLR stimulation in mouse pDCs. However, such conflicts may be explained by different technical approaches. Indeed, Lamagna *et al*.^28^ used total BM cultures (containing pDCs and cDCs) and/or CpG accomplished with the liposomal transfection reagent DOTAP, which could override the SFK requirement by altering normal CpG internalization or trafficking^17^. Indeed, we observed that SFK action in pDCs was dispensable for TLR ligand overall uptake, but was necessary for optimal trafficking to early endosomes. Remarkably, CpG trafficking to lysosomes in cDCs was not affected by SFK inhibition, implying different SFK requirements for TLR-ligand localization in pDC versus cDCs. Although further investigation is necessary, it is tempting to speculate that such different SFK-dependent and -independent internalization mechanisms among pDCs and cDCs, respectively, could partly underlie their distinct TLR-ligand organelle localization and downstream responses^17^. SFKs have been described to be involved in a multitude of internalization processes^49^, but many studies have been done focusing on SRC, a nonpalmitoylated kinase reported to have a different subcellular compartmentalization than palmitoylated SFKs, such as FYN and LYN^50^. Indeed, SRC is important for formation and trafficking of macropinosomes, whereas palmitoylated SFKs are not^51^.

Remarkably, we also observed that SFK activity was necessary to promote TLR-induced phosphorylation of IKKα/β, p38, NF-κB and IRF-7, whereas there was no role for the type I IFN-mediated STAT1 phosphorylation. These observations suggest that SFKs affect pDC activation through regulation of the TLR receptor signalling pathway but are not involved in the positive feedback loop driven by the type I IFNs produced early after stimulation^31^,^32^. In addition, we observed that SFKs were necessary to promote phosphorylation of MTOR, which favours constitutive IRF-7 expression and is required for nuclear translocation, thereby promoting type I IFN production^34^,^52^. Our results also indicated that SFK activity is necessary for BCAP tyrosine phosphorylation, but it is still unclear if this event was directly mediated by SFKs or was secondary to the activity of other kinases downstream SFKs, such as SYK or BTK, which, similarly to LYN, have been suggested as possible candidates for BCAP tyrosine phosphorylation^37^,^38^. Studies in macrophages reported BCAP as an adaptor protein interacting with MYD88 and PI3K, recruiting the latter through a phosphorylated tyrosine, and thereby linking TLR activation to PI3K pathway^35^,^36^. PI3K has been shown to be critical for type I IFN production by pDCs in response to TLR^33^, and, consistently, BCAP has been recently suggested as a positive regulator of TLR-mediated pDC activation^53^. Based on these considerations, we hypothesized that the reduced MTOR activation that we observed when SFKs were inhibited could have been, at least partially, related to a defective activation of the PI3K-AKT-MTOR pathway due to impaired BCAP phosphorylation.

Interestingly, we detected SFK-dependent BCAP tyrosine phosphorylation even in the absence of TLR stimulation, consistent with the possibility that SFKs might be involved in a tonic signal necessary to induce a ready-to-respond state and ensure a rapid response by pDCs. This notion is also supported by the fact that pDCs show constitutive MTOR phosphorylation even in the absence of external stimulation^34^. In addition, we previously showed that CD28 exerts a negative regulatory role in mouse pDCs and this might also take place before pathogen encounter^54^. The need for a basal signalling tuning the cellular response to external stimulation has been already theorized and confirmed for many other immune cell types, such as macrophages, where a tonic ITAM signalling by DAP12 amplifies interleukin-10 and IFN-α-induced STAT1-dependent gene expression^55^, and B cells, where a continued antigen-independent ITAM-mediated B-cell receptor signalling has a physiological role in B-cell development and survival^56^. Notably, these and other basal signals involve immunoreceptors and kinases belonging to both the Src family and the Syk family, and might be similar to the one ongoing in pDCs^57^. Although there may be multiple yet-to-be-discovered receptors engaging SFK signalling to prime pDCs independently of TLR-ligand stimulation, it is possible that CD4 and/or CD8, which can be expressed in pDCs^12^ and recruit LCK and FYN in T cells^58^, may also engage SFKs in pDCs. This likely brings activated SFKs in close proximity to ITIM-bearing receptors, which positively regulate pDC responses^59^. Of note, Ly49Q is an ITIM-bearing receptor in mouse pDCs that binds to its ligand H2-K^b^ in steady-state conditions and is essential for efficient CpG redistribution with TLR9 and type I IFN production^60^.

Bafetinib is a dual BCR-ABL/LYN inhibitor developed as a therapeutic against Philadelphia-positive leukaemia and has been already proved safe for use in humans^42^,^43^. A kinase inhibition profile, in which 79 tyrosine kinases were tested, showed that bafetinib is able to almost entirely and selectively inhibit the activity of ABL, ABL-related gene and LYN^41^. In addition, it also inhibits more than 75% of the FYN activity and almost 50% of the BLK activity. Instead, it is not able to inhibit SRC and YES, the other SFKs tested. We observed that treatment of CAL-1 and blood-derived primary human pDCs with bafetinib caused a profound and dose-dependent reduction of TLR-mediated type I IFN and TNF production. Remarkably, the highest bafetinib dosage tested reduced the type I IFN production to undetectable levels. Furthermore, we observed that bafetinib behaved similarly to PP2, inhibiting key elements downstream TLR signalling including NF-κB, IRF-7, p38 and MTOR but without affecting STAT1-dependent type I IFN-positive feedback loop.

Our study contributes to understand the mechanisms tuning mice and human pDC responses, identifying SFK members Fyn and Lyn as positive regulators of TLR-induced type I IFN and pro-inflammatory cytokine production. In addition, our findings support a model in which pDCs constitutively engage SFK members Fyn and Lyn to trigger a tonic signalling pathway that enables their optimal response to subsequent TLR stimulation. Given the important roles of pDCs in host defence against pathogens, tumour immune-surveillance, wound healing and autoimmune diseases^3^,^6^,^7^,^14^,^44^,^45^,^61^,^62^, manipulation of selected SFK activity in pDCs, perhaps with small molecules such as bafetinib coupled to pDC-specific antibodies, could bring about novel therapeutic strategies for several human illnesses.

## Methods

### Animals and virus

Wild-type (WT) C57Bl/6 and Fyn knockout (Fyn^−/−^) mice were purchased from The Jackson Laboratory (Bar Harbor, ME, USA). Lyn knockout (Lyn^−/−^) were a gift from Dr Kawakami T. (La Jolla Institute for Allergy and Immunology, San Diego, CA, USA) and their origins have been described by Chan *et al*.^63^. Mice were bred and maintained in a closed breeding facility, and mouse handling complied with the requirements of the National Institutes of Health and received approval from the Institutional Animal Care and Use Committee of the University of California San Diego. Mice were infected i.p. with 1 × 10^4^ plaque-forming unit of MCMV Smith strain. MCMV Smith strain was prepared by *in vivo* propagation in 3-week-old BALB/c mice infected with 1 × 10^4^ plaque-forming unit by i.p. Salivary gland homogenates were prepared at day 14 post-infection, and viral titre was determined by plaque assay. Six- to twelve-week-old mice from both sexes were used for the experiments. No randomization was used, and no blinding was done. No statistical method was used to estimate animal sample size.

### Antibodies

For flow cytometry and confocal microscopy, the following antibodies were used: anti-mouse Thy1.2 (clone 30-H12, 1:400), anti-mouse CD11c (N418, 1:100), anti-mouse B220 (RA3-6B2, 1:100), anti-mouse CD8a (53-6.7, 1:200), anti-mouse F4/80 (BM8, 1:200), anti-human CD3 (UCHT1, 1:200), anti-human CD14 (HCD14, 1:200), anti-human CD16 (3G8, 1:200), anti-human CD19 (HIB19, 1:200), anti-human CD56 (MEME-188, 1:200), anti-human CD11c (3.9, 1:50), anti-human CD123 (6H6, 1:50), anti-human CD304 (12C2, 1:50), anti-human HLA-DR (L243, 1:50), anti-human TNF-α (Mab11, 1:50) from Biolegend (San Diego, CA, USA); anti-mouse CD19 (eBio1D3, 1:400), anti-mouse NK1.1 (PK136, 1:400), anti-mouse CD11b (M1/70, 1:200), anti-mouse CD317 (eBio927, 1:300), anti-human p-MTOR (MRRBY, 1:50) from eBioscience (San Diego, CA, USA); anti-mouse EEA1 (Clone 14, 1:100), anti-human p-STAT1 (4a, 1:50), anti-human p-NF-κB (K10-895.12.50, 1:25) from BD Biosciences (San Jose, CA, USA); anti-human IFN-α (LT27:295, 1:10); anti p-IRF-7 (REA310, 1:30) from Miltenyi Biotec (Auburn, CA, USA); anti-mouse LAMP2 (Clone GL2A7, 1:100) from Developmental Studies Hybridoma Bank (DSHB) at the University of Iowa; anti-human p-p38 (D3F9, 1:200) from Cell Signaling Technology (Danvers, MA, USA). For immunoprecipitation and immunoblotting, the following antibodies were used: anti-phospho Src family Tyr-416 (D49G4, 1:5,000), anti-human p-IKKα/β Ser176/180, anti-human FYN, anti-human NF-κB (D14E12, 1:5,000), anti-human IκBα (L35A5, 1:5,000), anti-human AKT, anti-human p-AKT Ser473, anti-human p-MTOR Ser-2448, anti-human MTOR from Cell Signaling Technology; anti-human IKKα/β (H-470, 1:2,000) and anti-human FYN (E-3, 1:1,000) from Santa Cruz Biotechnology (Santa Cruz, CA, USA); anti-human LYN (LYN-01, 1:1,000) from Biolegend; anti-human PIK3AP1 from Abcam; anti-phospho tyrosine (4G10, 1:1,000) from EMD Millipore (Temecula, CA, USA).

### Cell purification

Mouse spleens were incubated with Collagenase D (1 mg ml^−1^, Roche, Indianapolis, IN, USA) for 20 min at 37 °C, passed through a 100 μm strainer to obtain a single-cell suspension, and subsequently FACS purified using a BD ARIA (BD Biosciences) for B cells (CD19^+^), T cells (Thy1.2^+^), macrophages (Thy1.2^−^CD19^−^NK1.1^−^CD11c^−^CD11b^+^F4/80^+^), pDCs (Thy1.2^−^CD19^−^NK1.1^−^CD11c^+^CD11b^−^B220^+^PDCA^+^) and cDCs (Thy1.2^−^CD19^−^NK1.1^−^ and CD11c^+^B220^−^CD11b^+^ or CD11c^+^B220^−^CD8^+^).

BM cells were isolated from femurs and tibias and incubated in RPMI with 10% foetal bovine serum (Lonza, Walkersville, MD, USA) containing 100 ng ml^−1^ of Flt3L (Amgen, Thousand Oaks, CA, USA) for 8 days. At day 8, BM-derived DC (BMDCs) were FACS purified for pDCs (CD11c^intermediate/dim^CD11b^−^B220^+^PDCA^+^) and CD11b^+^ cDCs (CD11c^+^B220^−^CD11b^+^CD8^−^). Purity for all cell types was >95%.

### Primary human cells

PBMCs were isolated from whole blood obtained from healthy volunteers using Ficoll-Paque PLUS (GE Healthcare). Enrichment of pDCs was performed using the Diamond Plasmacytoid Dendritic Cell Isolation kit (Miltenyi Biotec). The UCSD Institutional Review Board certified the study as not human subject research, thereby informed consent was not required. The activities associated with the project are conducted in compliance with applicable UCSD and Rady Children's Hospital—San Diego policies and ethical standards as well as local, state and federal regulations.

### Culture and cell line maintenance

CAL-1 cell line was provided by Dr S. Kamihira (Nagasaki University Graduate School of Biomedical Sciences, Nagasaki, Japan). CAL-1 cell line was cultured in RPMI 1640 (Lonza, Walkersville, MD, USA), supplemented with 2 mM L-glutamine, 50 U ml^−1^ penicillin and 50 μg ml^−1^ streptomycin (Gibco, Grand Island, NY, USA), plus 10% heat-inactivated foetal bovine serum (Lonza). LYN-deficient CAL-1 were generated by CRISPR/Cas9-mediated genome engineering following the protocol described by Ran *et al*.^64^. A target sequence in the fourth exon of human LYN (5′-GTAGCCTTGTACCCCTATGATGG-3′, PAM motif underlined) was chosen and appropriate oligonucleotides were cloned into the BbsI site of pSpCas9(BB)-2A-GFP plasmid obtained from the laboratory of Feng Zhang via Addgene (Cambridge, MA, USA; plasmid ID: 48138). The plasmid was introduced into CAL-1 using the Amaxa human monocyte nucleofector kit (Lonza), and 1 day later the reporter-positive population was sorted and plated as a single-cell suspension in ClonaCell-TCS Medium (StemCell Technologies, Vancouver, CA, USA) for semi-solid cloning. One week later, clonal cells were collected, expanded, tested for LYN expression and maintained in 175 cm^2^ flasks at a density of 1–2 × 10^6^ cells ml^−1^ in a total volume of 30 ml.

### Genomic DNA isolation and sequencing

Genomic DNA was isolated from CAL-1 and CRISPR/Cas9-treated CAL-1 using QIAmp DNA mini kit (Qiagen, Valencia, CA, USA). Extracted DNA were subjected to PCR to amplify the region near the designed CRISPR guide, using primers described in Supplementary Table 1. Amplified product was gel purified using QIAquick Gel Extraction Kit (Qiagen) and sent to Eton Bioscience (San Diego, CA, USA) to be Sanger sequenced.

### Nucleic acid isolation and RT-qPCR

Cell lines or primary purified cells were collected in lysis buffer (Qiagen). RNA was purified using RNeasy extraction kits with a DNase (Qiagen) incubation step to digest any trace genomic DNA present. For RNA extraction from cell line, 5 × 10^5^ cells were lysed and RNA extracted using RNeasy mini columns, and for primary cells, 3–10 × 10^4^ cells were lysed and RNA extracted using RNeasy micro columns. For standard qPCR analysis of relative mRNA expression levels, cDNA was synthesized using SuperScript III (Life Technologies, Carlsbad, CA, USA) followed by incubation with RNase H according to the manufacturer's protocol. All cDNA products were stored at −20 °C. Quantification of cDNA was performed using Fast SYBR Green PCR Kit or TaqMan Fast Universal PCR kit (Life Technologies). The relative RNA levels were normalized against *Gapdh* RNA. Primer sequences are reported in Supplementary Table 1.

### Cytokine measurements

For cytokine measurement in culture supernatants, 1 × 10^6^ total BMDCs, 5 × 10^4^ FACS-purified pDCs, 1 × 10^5^ CAL-1 or 3 × 10^6^ PBMCs were left untreated or pre-treated for 1 h with the SFKs inhibitor PP2 (EMD Millipore), the inhibitor bafetinib (Adooq, Irvine, CA, USA) or the equivalent concentration of DMSO control, followed by 15 h in media in presence or absence of 0.1 μM CpG-B ODN 1668 (Roche), 1 μM CpG-A ODN 2216, 100 μM loxoribine (mouse cells) or 1 μg ml^−1^ R848 (human cells) (all from InvivoGen, San Diego, CA, USA).

Mouse type I IFN bioactivity was measured with reference to a recombinant mouse IFN-β standard (Research Diagnostics, Concord, MA, USA) using a L-929 cell line transfected with an interferon-sensitive luciferase. Human type I IFN bioactivity was measured with reference to a recombinant human IFN-β standard (InvivoGen) using HEK-Blue IFN-α/β cell line (InvivoGen). Mouse serum IFN-α, mouse and human TNF were measured, respectively, by luminescent ELISA (LumiKine mIFN-α, InvivoGen) or by ELISA (Mouse TNF-α ELISA Ready-SET-Go!, eBioscience, and Human TNF-α ELISA MAX, Biolegend) as described in the manufacturer's protocol.

### Flow cytometry

Cells were pre-incubated with CD16/CD32 Fc block (BD PharMingen). Surface and intracellular cytokine staining was performed per manufacturer's instructions. PI or Ghost dye (Tonbo Biosciences, San Diego, CA, USA) was used to exclude dead cells and to measure cell viability where indicated. Phosflow staining was performed fixing the cells with BD Phosflow Lyse/Fix Buffer 5 × for 10 min at 37 °C, and then permeabilizing with BD Phosflow Perm Buffer III (BD Biosciences) for intracellular staining with Abs for phospho-proteins. Samples were acquired on a BD LSR II (BD Biosciences) and analysed using FlowJo software (Treestar, Inc., Ashland, OR, USA).

### Immunoprecipitation and immunoblotting

CAL-1 cells were pre-treated with either PP2 or DMSO control for 1 h, stimulated with R848 (1 μg ml^−1^) and subsequently lysed with immunoprecipitation lysis buffer (Thermo Scientific, Rockford, IL, USA) supplemented with protease inhibitor cocktail set III (EMD Millipore) and phosphatase inhibitor cocktail set I and II (EMD Millipore) for immunoprecipitation analysis, with RIPA buffer (Thermo Scientific) supplemented with protease inhibitor cocktail set III (EMD Millipore) for immunoblotting analysis or processed with the Subcellular Protein Fractionation Kit for Cultured Cells (Thermo Scientific) for subcellular fractionation. For immunoprecipitation, 1 mg of each lysate was incubated on a rotator for 1 h at 4 °C with the appropriate amount of the respective antibody. An aliquot of 30 μl of Protein G Sepharose Fast Flow (Sigma, St Louis, MO, USA) were added, followed by 2 h incubation on a rotator at 4 °C, and immunoprecipitated proteins were recovered with an equal amount of 4 × laemmli sample buffer (Bio-Rad, Hercules, CA, USA) containing 2-ME. Proteins were resolved by 8, 10, 12% SDS–PAGE, were electrophoretically transferred to PVDF membranes (EMD Millipore) and blocked with 3% BSA, 5% BSA or 5% non-fat dry milk (according to the primary antibody) in Tris-buffered saline containing 0.1% Tween-20; they were then probed with the desired primary antibody and an appropriate HRP-conjugated antibody and eventually visualized by ECL Plus (Thermo Scientific). When necessary, membranes were stripped using Restore Plus Western Blot Stripping Solution (Thermo Scientific).

### CpG uptake and trafficking

For analysis by flow cytometry, BM-derived DCs were pre-treated with either PP2 or DMSO control for 1 h and then stimulated with CpG-A-FITC 3 μM (Invivogen) or CpG-B-FITC 3 μM (Invivogen) for 1 h. Cells were then PBS washed, treated with trypsin 0.1% to decrease background signal from unspecific surface binding, fixed with paraformaldehyde 1% and then surface stained as described above.

For confocal microscopy, BM-derived DCs plated on Poly-D-lysine coated coverslips were treated and stimulated as above for 90 min. Cells were fixed at room temperature with 3% paraformaldehyde for 20–25 min, permeabilized (0.2% Triton X-100) for 45 min and incubated for 1 h each with primary and then secondary antibodies. Images were acquired using a Leica CTR4000 Confocal Microscope with a × 63 objective. To estimate the degree of colocalization in immunofluorescence assays, ImageJ RGB Profiler plug-in was used to determine the intensity fluorescence distribution for corresponding fluorophore (green and red channels). Red–green–blue (RGB) graphic profiles were created by analysing the distribution and intensity of pixels of these colours along a chosen line using ImageJ software. All individual images were processed using ImageJ software and assembled for presentation using Photoshop and Illustrator software (Adobe).

### Statistical analysis

Statistical differences were determined by Student's *t*-test, Mann–Whitney test, *χ*^2^-squared test or, when comparing more than two sets of values, by one-way or two-way analysis of variance (ANOVA) followed by Dunnett *post-hoc* analysis using the GraphPad Prism 5 software (La Jolla, CA, USA).

### Data availability

The data that support the findings of this study are available from the corresponding author on reasonable request.

## Additional information

**How to cite this article:** Dallari, S. *et al*. Src family kinases Fyn and Lyn are constitutively activated and mediate plasmacytoid dendritic cell responses. *Nat. Commun.* **8,** 14830 doi: 10.1038/ncomms14830 (2017).

**Publisher's note:** Springer Nature remains neutral with regard to jurisdictional claims in published maps and institutional affiliations.

## Supplementary Material

Supplementary Information Supplementary Figures and Supplementary Table
