# Supplementary material for: Src family kinases Fyn and Lyn are constitutively activated and mediate plasmacytoid dendritic cell responses
Source: Nat Commun. 2017 Apr 3;8:14830. doi: 10.1038/ncomms14830 (PMC5382270; doi:10.1038/ncomms14830)
Supplement: Supplementary Information — Supplementary Figures and Supplementary Table [file ncomms14830-s1.pdf]

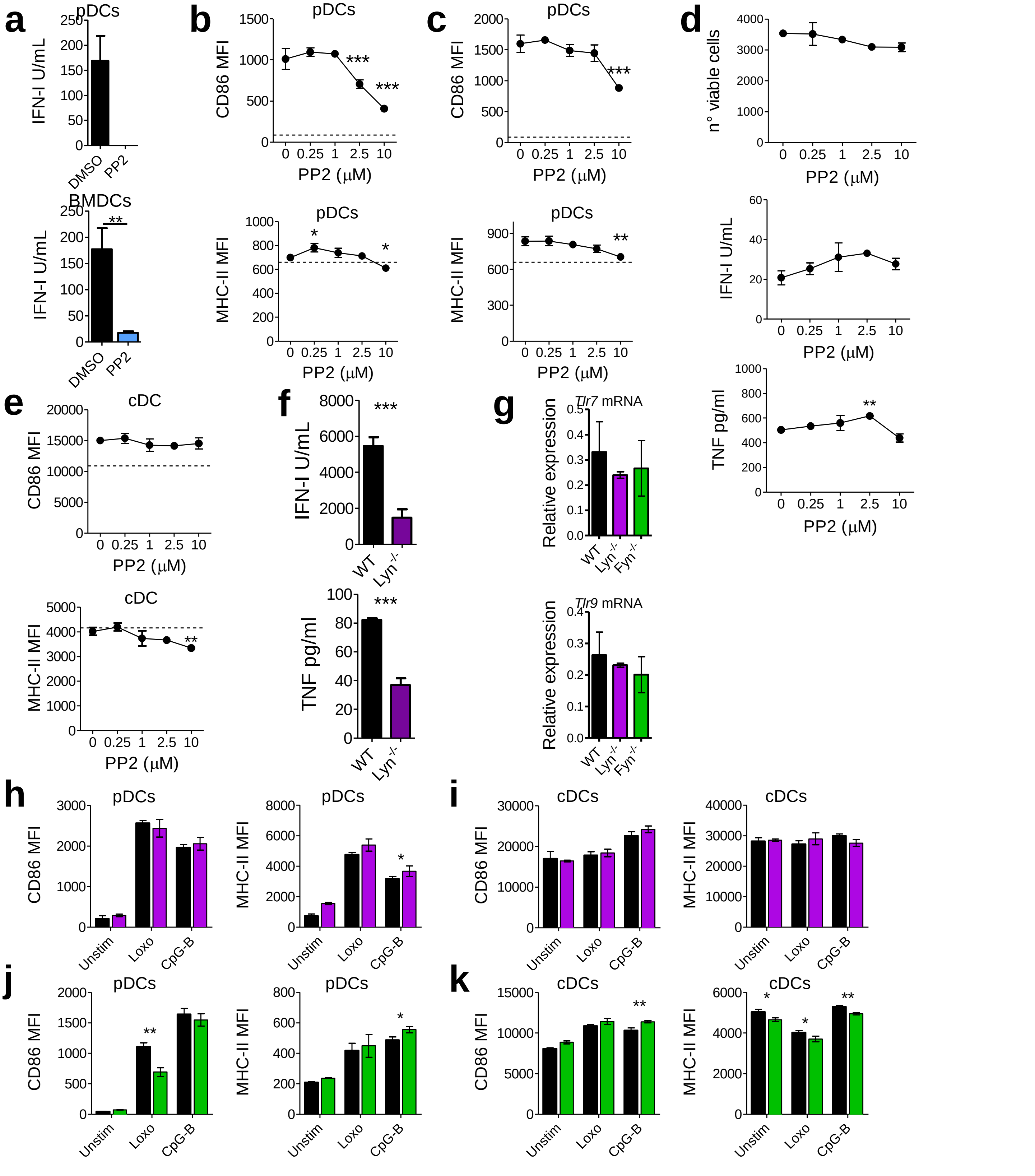

Supplementary Figure 1: SFK role in TLR-mediated upregulation of maturation markers in murine DCs

a-e) Total BM-derived DCs (**a**), FACS-purified BM-derived pDCs (**a-c**) or cDCs (**d, e**) were pre-treated with 10 µM PP2 (**a**; blue) or DMSO control (black) for 1 hour or left untreated (**b-e**) and then stimulated with CpG-A (**a**), CpG-B (**b, d, e**) or loxoribine (loxo) (**c**) for 15 hours. Surface expression of the activation markers CD86 and MHC-II were evaluated by flow cytometry (**b, c, e**). Cell viability was assessed by flow cytometry (**d**), while IFN-I and TNF protein levels in the supernatant were assessed by bioassay (**a, d**) and ELISA (**d**) respectively. **f)** FACS-purified BM-derived pDCs from WT (black) and Lyn$^{-/-}$ (violet) mice were stimulated with CpG-A for 15 hours. IFN-I and TNF protein levels in the supernatant were assessed by bioassay and ELISA, respectively. **g)** RNA from FACS-purified BM-derived pDCs from WT (black), Lyn$^{-/-}$ (violet) or Fyn$^{-/-}$ (green) were extracted and levels of Tlr7 and Tlr9 mRNA were assessed by quantitative PCR. **h-k)** FACS-purified BM-derived pDCs (**h, j**) and cDCs (**i, k**) from WT (**h-k**; black), Lyn$^{-/-}$ (**h, i**; violet) or Fyn$^{-/-}$ (**j, k**; green) mice were stimulated with CpG-B or loxoribine for 15 hours. Surface expression of CD86 and MHC-II were evaluated by flow cytometry. Data are representative of 2 (a-e, g) independent experiments or more than 3 repeats with n=2 pooled mice per group (f, h-k). Graphs depict mean ± SD of replicates within one representative experiment. Two-way Student's t-test (a, f-k) and Dunnett's multiple comparisons test (b-e) were used for statistical analysis. * p<0.05, ** p<0.01, ***p<0.001.

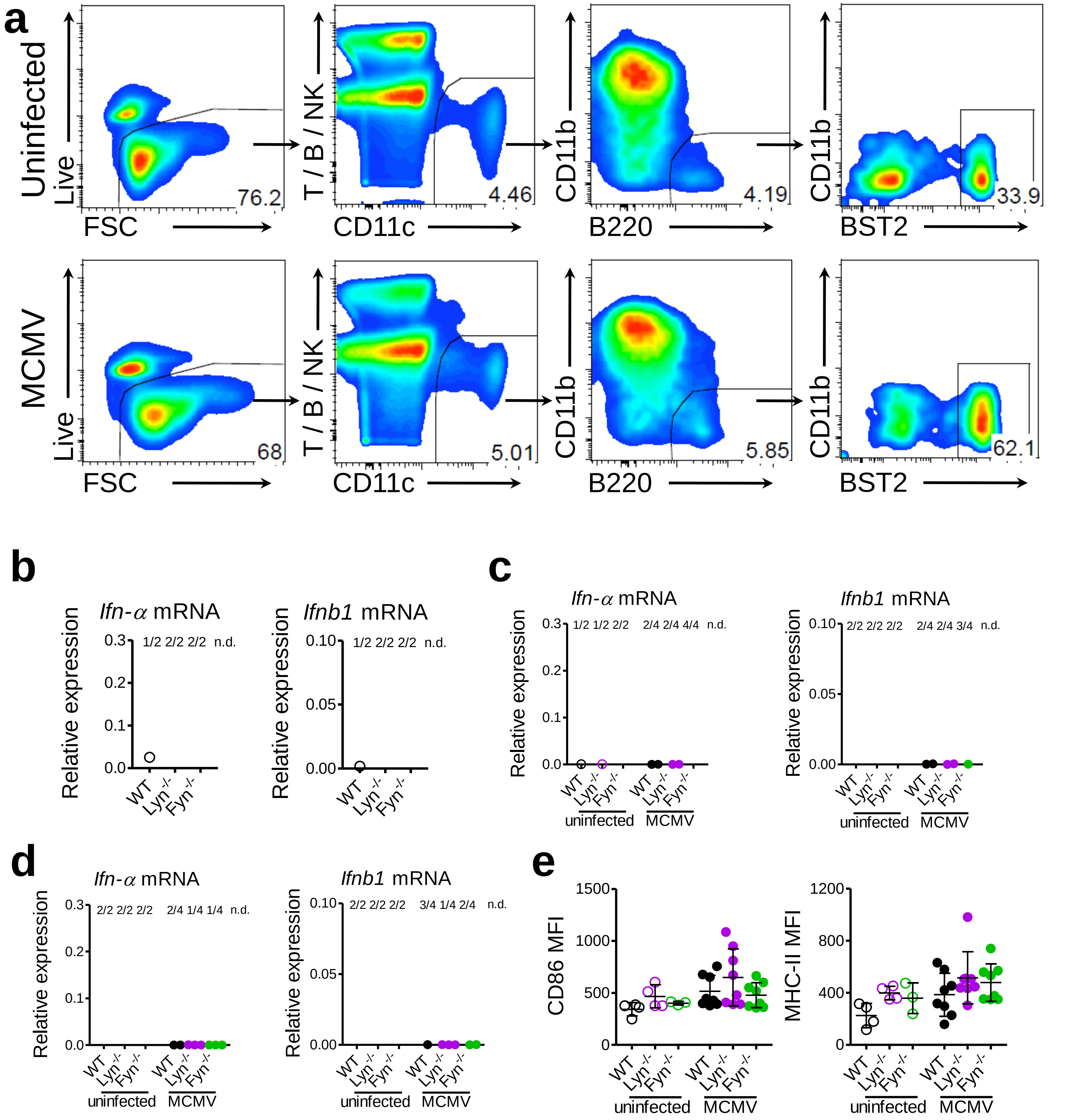

Supplementary Figure 2: Fyn and Lyn are dispensable for pDC upregulation of maturation markers after MCMV infection

a) Gating strategy for pDC identification in murine spleens. Cell duplets were excluded based on size. **b-d**) The levels of *Ifna* and *Ifnb1* were determined by q-PCR in uninfected (**b-d**) and 36 h MCMV infected (**c, d**) FACS purified slenic pDCs (**b**), cDCs (**c**) and macrophages (**d**). The y axis ranges are the same as in Figure 3b to facilitate the comparison. **e**) pDC expression of CD86 and MHC-II was evaluated by FACS in spleen mononuclear cells from mice infected with MCMV 36 h earlier or uninfected controls. Data are representative of two independent experiments with four mice per group. Kruskal-Wallis test was used for statistical analysis (e). n.d.: not detected. * $p<0.05$, ** $p<0.01$, *** $p<0.001$.

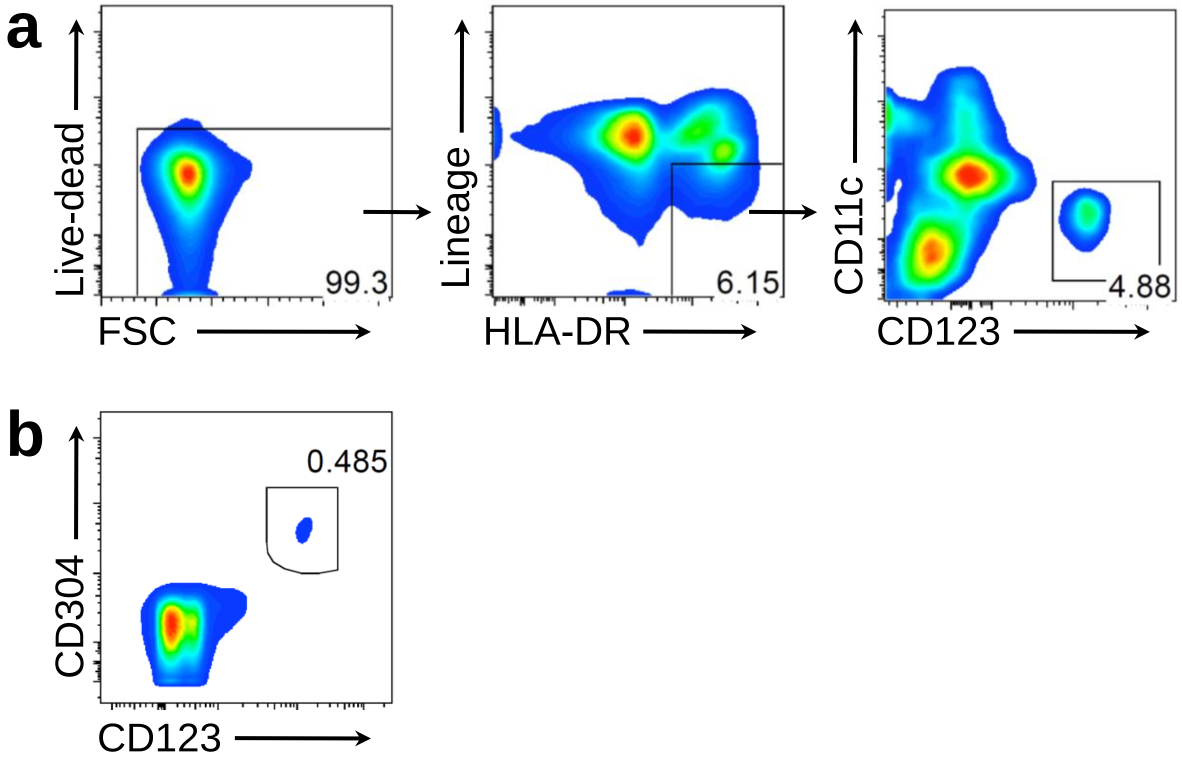

Supplementary Figure 3: Gating strategy for pDC identification in human peripheral blood
Gating strategy for pDC identification in human PBMCs under methanol-free (**a**) and methanol-based (**b**) staining protocol. Cell duplets were excluded based on size.

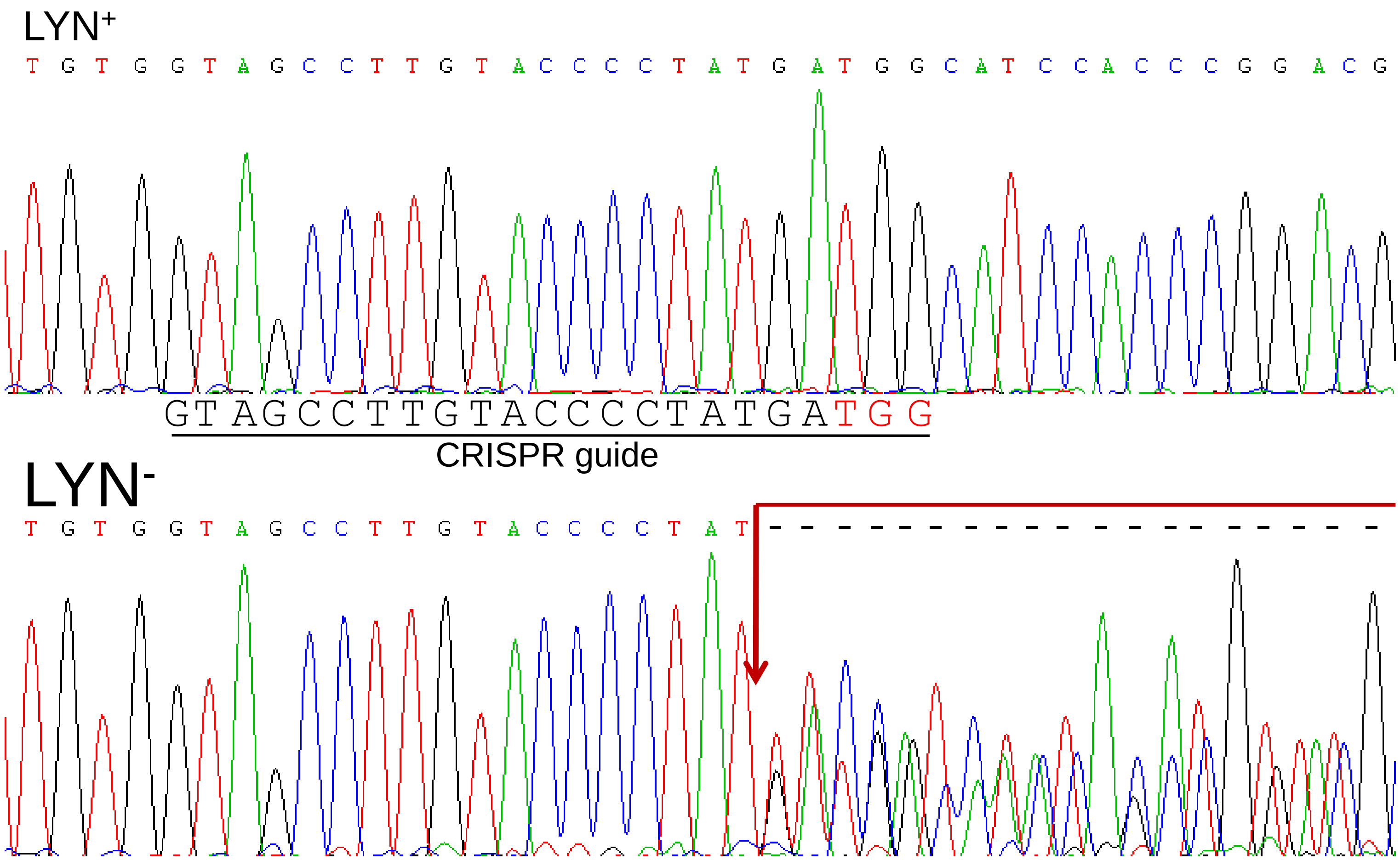

Supplementary Figure 4: Disruption of LYN gene by CRISPR/Cas9 technique

A portion of the genomic DNA of LYN+ and LYN- CAL-1 cell lines surrounding the sequence targeted by the CRISPR/Cas9 guide was amplified by PCR. The products were gel purified and Sanger sequenced. The arrow points from where the LYN genomic DNA sequence is disrupted in the LYN- cell line.

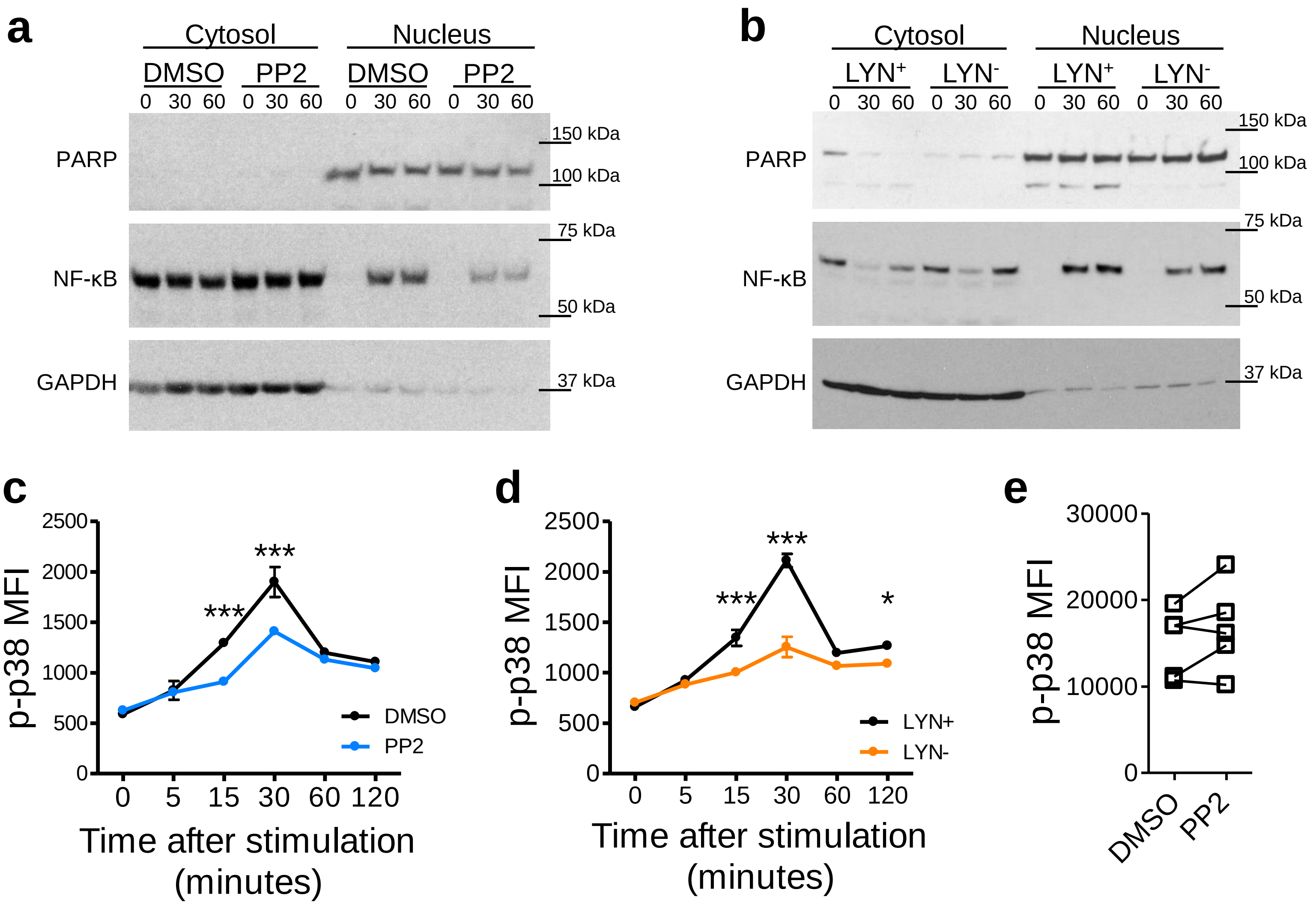

Supplementary Figure 5: SFKs are necessary for NF-κB nuclear translocation and P38 activation in CAL-1 cells

a, b) WT (**a**) or LYN$^-$ (**b**) CAL-1 cells were pre-treated for 1 hour with PP2 (10 μM) or DMSO control or left untreated before stimulation with R848 for the indicated time periods (minutes). In **a**, protein were fractionated into cytosolic and nuclear fraction and levels of PARP, NF-κB and GAPDH were determined by immunoblot. **c, d**) WT (**c**) or LYN$^-$ (**d**) CAL-1 cells were pre-treated for 1 hour with PP2 (10 μM; **c**; blue) or DMSO control (**c**; black) or left untreated (**d**; black: LYN$^+$, orange: LYN$^-$), before stimulation with R848 for the indicated time periods (minutes). Level of p-p38 was determined by flow cytometry. **e**) Human PBMCs were pre-treated as in **c** and stimulated for 30 min with R848. p-p38 was determined by flow cytometry in gated pDCs. Lines in graphs connect the same donor. Data are representative of 2 (a, b, d) or 3 (c) independent experiments or 5 donors processed separately (e). Graphs depict mean ± SD of replicates within one representative experiment (a-d) or individual donors (e). Two-way ANOVA (c, d) and Student's t-test (e) were used for statistical analyses. * $p<0.05$, ** $p<0.01$, *** $p<0.001$.

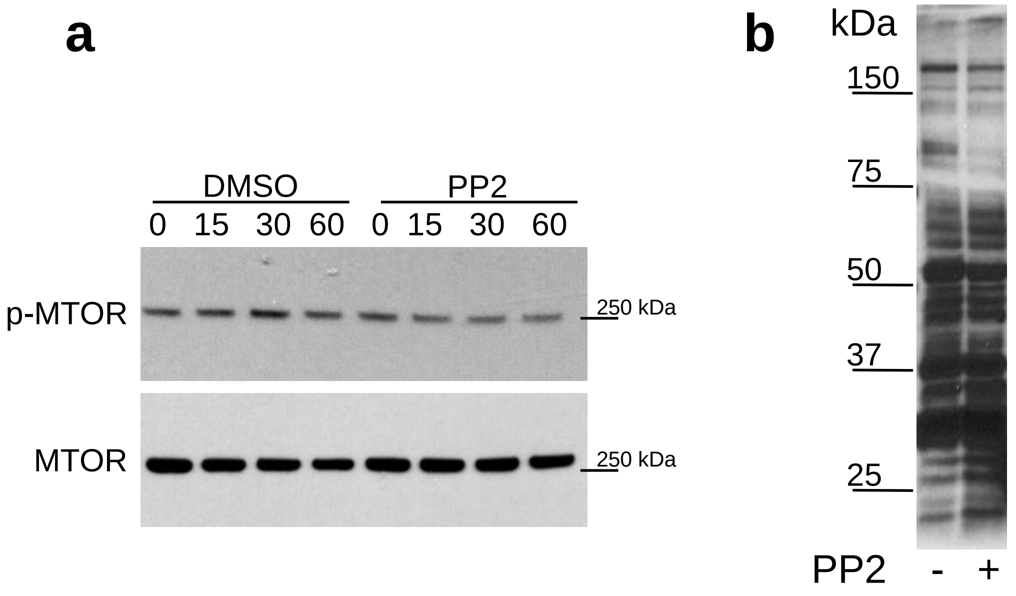

<u>Supplementary Figure 6: PP2 treatment decreases MTOR phosphorylation without leading to major changes in total tyrosine phosphorylation level in CAL-1 cells</u>

a) CAL-1 cells were pre-treated for 1 hour with PP2 (10 µM) or DMSO control before stimulation with R848 for the indicated time periods (minutes). p-MTOR and MTOR levels were determined by immunoblot. **b**) CAL-1 cells were pre-treated for 1 hour with PP2 (10 µM) or DMSO control. After, cells were harvested and protein lysates were collected. The phosphorylated tyrosine level was assessed by immunoblot. Results are representative of 2 (a) or 5 (b) independent experiments.

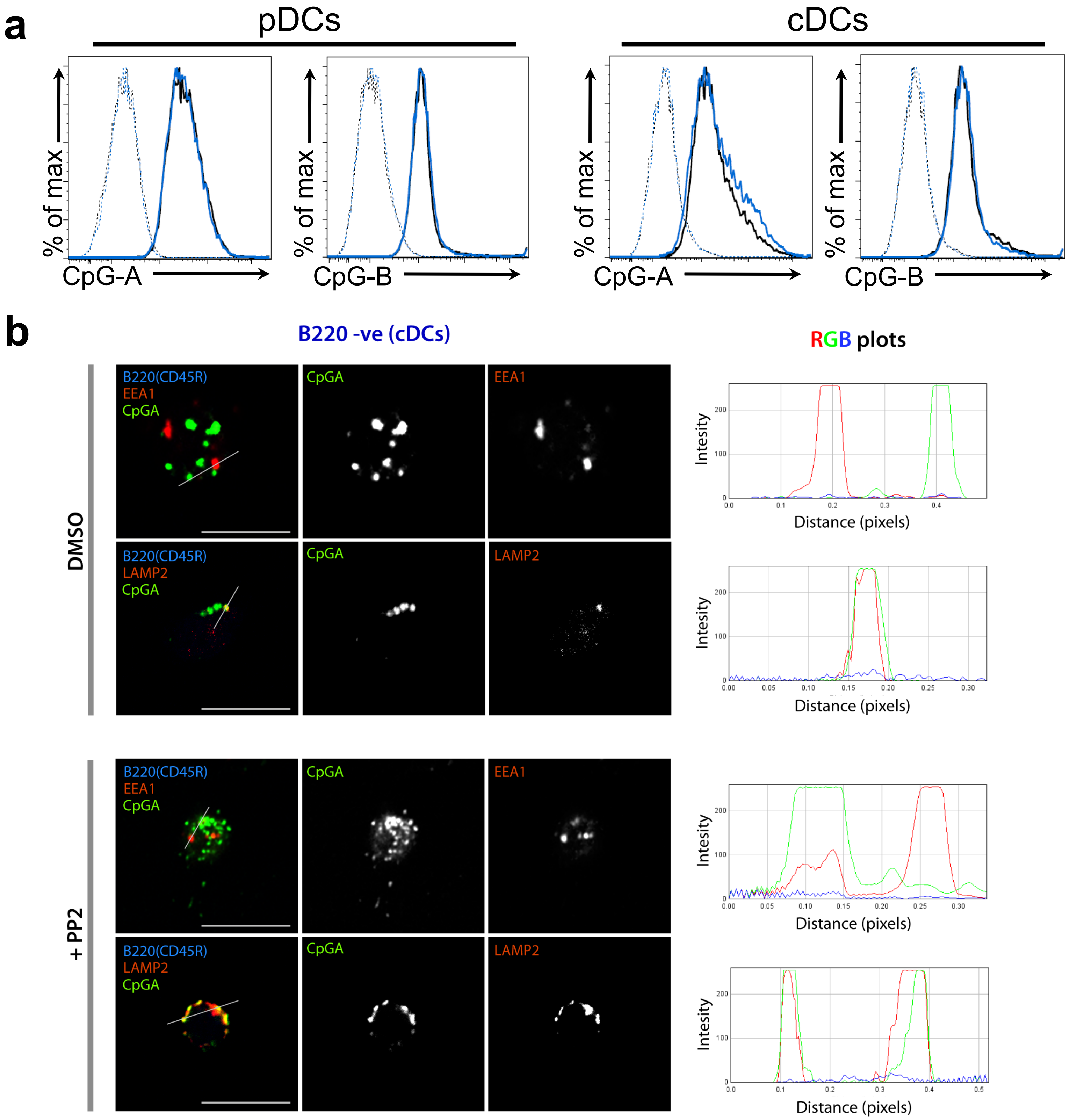

Supplementary Figure 7: SFK activity is dispensable for overall CpG update in pDC and cDCs and TLR ligand localization in cDCs.

a) BM-derived DCs pre-treated for 1 hour with PP2 (10 μM; blue) or DMSO control (black) and then stimulated with either CpG-A-FITC or CpG-B-FITC for 1 hour. Histograms depicts 0 minutes (dashed lines) and 60 minutes (solid lines) time-points analyzed by flow cytometry. pDCs were gated as $CD11c^{+}/CD11b^{-}/BST2^{+}$. **b**) Confocal images of $B220^{-}$ BM-derived DCs pre-treated for 1 hour with PP2 (10 μM) or DMSO control and then stimulated with CpG-A-FITC for 90 minutes. RGB plots indicate the degree of colocalization of CgG-A-FITC (green) with EEA1 or LAMP2 (red) along the arbitrary line on each corresponding merged image panel, as measured using ImageJ. Images are representative of 2 (**b**) or 3 (**a**) independent experiments. Scale bar represent 10 μm. * p<0.05, ** p<0.01, ***p<0.001.

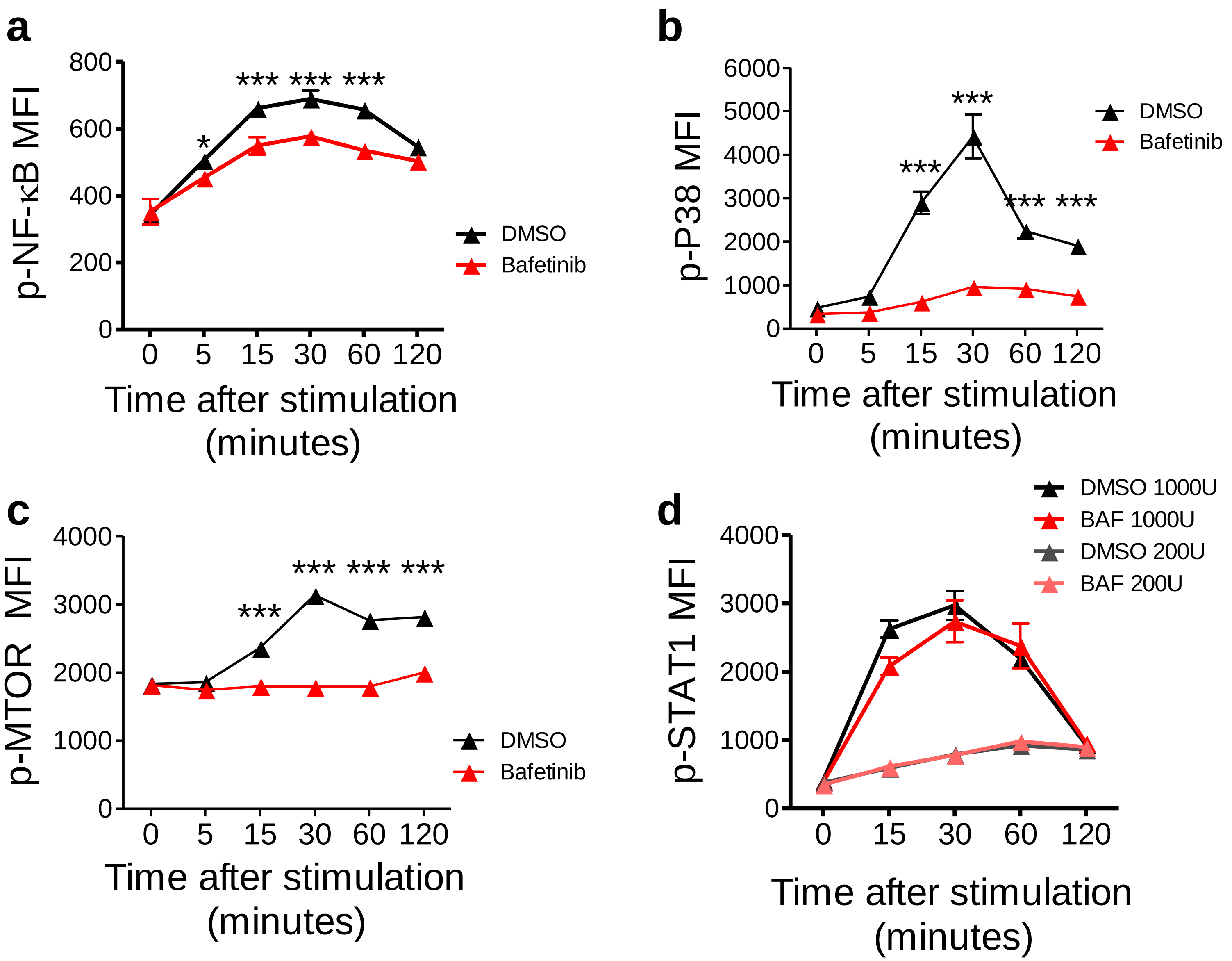

Supplementary Figure 8: Bafetinib inhibits TLR-7 but not IFNAR signaling in CAL-1 cells

a-d) CAL-1 cells were pre-treated for 1 hour with bafetinib (5 μM) or DMSO control and then stimulated with R848 (A-C) or different amount of rIFN-β (**d**) for the indicated time periods (minutes). p-NF-κB (**a**), p-p38 (**b**), p-MTOR (**c**), p-STAT1 (**d**) were determined by flow cytometry. Data are representative of 2 (**d**) or 5 (**a-c**) independent experiments. Graphs depict mean ± SD of replicates within one representative experiment. Two-way ANOVA was used for statistical analysis. * $p<0.05$, ** $p<0.01$, *** $p<0.001$.

	Primer Forward	Primer Reverse
LYN amplification for sequencing	CAGCAAGGTACTCTCTGTGGG	GGGGTTGAGGAGATGACGAC
Murine Blk	AAGCCACTGAAGCTGACTGAGA	TCACTGACCTGCCTCTTGCTG
Human BLK	CCTGCTCCACTGTAAGGGTG	TCCATTGGCCCTTGTCCTTC
Murine Fgr	AGATCCGAAAGCTGGACACG	AGAGTCTGGGGCTTAGTGGT
Human FGR	CCGGAAAAACTGGCTGCATT	GGTCTCGCTTTCCCGAATGA
Murine Fyn	AAGCACGGACGGAAGATGAC	ATGGAGTCAACTGGAGCCAC
Human FYN	ACAGCTCGGAAGGAGATTGG	CTGTCGCTCAGCATCTTTTCG
Murine Hck	GGCACGAATCATCGAGGACA	GTAAGGGATCCGGCCATAG
Human HCK	CCATCAAGTGGACAGCTCCT	AGCTCGGATCACTTCAGGGT
Murine Lck	TGGGACCTTCACCATCAAGTC	TGTCAGGTCTCACCATGCG
Human LCK	TCCTGCTGACGGAAATTGTCA	CACACTGCGCAGGTAGTCA
Murine Lyn	TGGCTAAGGGTAGTTTGCTGG	CGCAGATCACGGTGGATGTA
Human LYN	ACCAGGGAGGAGCCCATTTA	CTTCCGCTCGATGTATGCCA
Murine Src	GCCTCACTACCGTATGTCC	TTTTGATGGCAACCCTCGTG
Human SRC	GCTGTTCGGAGGCTTCAACT	CCAGTCTCCCTCTGTGTTGT
Murine Yes	TGAGGCTGCTCTGTATGGTC	GCATTCTGTATCCCCGCTCT
Human YES	GCTGCACTGTATGGTCGGTT	TCCTGTATCCTCGCTCCACT
Murine IFN-α	TATGTCCTCACAGCCAGCAG	TTCTGCAATGACCTCCATCA
Murine TNF-α	CCCTCACACTCAGATCATCTTCT	GCTACGACGTGGGCTACAG
Murine Isg15	GAGCTAGAGCCTGCAGCAAT	CTTCTGGGCAATCTGCTTCT
Murine Ddx58	AAGAGCCAGAGTGTCAGAATCT	AGCTCCAGTTGGTAATTTCTTGG
Murine Tlr7	ATGTGGACACGGAAGAGACAA	GGTAAGGGTAAGATTGGTGGTG
Murine Tlr9	ATGGTTCTCCGTCGAAGGACT	GAGGCTTCAGCTCACAGGG
Human IFN-β	AAACTCATGAGCAGTCTGCA	AGGAGATCTTCAGTTTCGGAGG
Human TNFα	ACCCACACCATCAGCCGCAT	TGGCCAGAACCAAAGGCTCCCT
Human GAPDH	TGATGACATCAAGAAGGTGGTGAAG	TCCTTGGAGGCCATGTGGGCCAT
Murine Gapdh (SYBR)	TCCCACTCTTCCACCTTCGA	AGTTGGGATAGGGCCTCTCTT
Murine IFN-β (Taqman)	CTGGCTTCCATCATGAACAA	GAGGGCTGTGGTGGAGAA
	Probe 18 from Universal Probe Library (Roche, Indianapolis, IN)	
Murine Gapdh (Taqman)	AGCTTGTCATCAACGGGAAG	TTTGATGTTAGTGGGGCTTCG
	Probe 9 from Universal Probe Library (Roche, Indianapolis, IN)	

<u>Supplementary Table 1: Primers used in this study</u>
